# Analysis of genes (*TMEM106B*, *GRN*, *ABCC9*, *KCNMB2*, and *APOE*) implicated in risk for LATE-NC and hippocampal sclerosis provides pathogenetic insights: a retrospective genetic association study

**DOI:** 10.1186/s40478-021-01250-2

**Published:** 2021-09-15

**Authors:** Adam J. Dugan, Peter T. Nelson, Yuriko Katsumata, Lincoln M. P. Shade, Kevin L. Boehme, Merilee A. Teylan, Matthew D. Cykowski, Shubhabrata Mukherjee, John S. K. Kauwe, Timothy J. Hohman, Julie A. Schneider, Alzheimer’s Disease Genetics Consortium, David W. Fardo

**Affiliations:** 1 Department of Biostatistics, College of Public Health, University of Kentucky, 201 Multidisciplinary Science Building, 725 Rose Street, Lexington, KY 40536-0082 USA; 2 Sanders-Brown Center On Aging and Alzheimer’s Disease Research Center, University of Kentucky, Lexington, KY 40536 USA; 3 Pathology and Laboratory Medicine, University of Kentucky, Lexington, KY 40536 USA; 4 Department of Biology, Brigham Young University, Provo, UT 84602 USA; 5 Department of Epidemiology, National Alzheimer’s Coordinating Center, University of Washington, Seattle, WA 98195 USA; 6 Department of Pathology and Genomic Medicine, Houston Methodist Hospital, Houston, TX 77030 USA; 7 Department of Medicine, University of Washington, Seattle, WA 98104 USA; 8 Vanderbilt Memory & Alzheimer’s Center, Department of Neurology, Vanderbilt University Medical Center, Nashville, TN 37232 USA; 9 Departments of Neurology and Pathology, Rush University Medical Center, Chicago, IL 60612 USA

**Keywords:** Dementia, Proteinopathy, Pleiotropy, Arteriolosclerosis, SNP, Mixed pathology

## Abstract

Limbic-predominant age-related TDP-43 encephalopathy neuropathologic change (LATE-NC) is the most prevalent subtype of TDP-43 proteinopathy, affecting up to 1/3rd of aged persons. LATE-NC often co-occurs with hippocampal sclerosis (HS) pathology. It is currently unknown why some individuals with LATE-NC develop HS while others do not, but genetics may play a role. Previous studies found associations between LATE-NC phenotypes and specific genes: *TMEM106B*, *GRN*, *ABCC9*, *KCNMB2*, and *APOE*. Data from research participants with genomic and autopsy measures from the National Alzheimer’s Coordinating Center (NACC; n = 631 subjects included) and the Religious Orders Study and Memory and the Rush Aging Project (ROSMAP; n = 780 included) were analyzed in the current study. Our goals were to reevaluate disease-associated genetic variants using newly collected data and to query whether the specific genotype/phenotype associations could provide new insights into disease-driving pathways. Research subjects included in prior LATE/HS genome-wide association studies (GWAS) were excluded. Single nucleotide variants (SNVs) within 10 kb of *TMEM106B*, *GRN*, *ABCC9*, *KCNMB2*, and *APOE* were tested for association with HS and LATE-NC, and separately for Alzheimer’s pathologies, i.e. amyloid plaques and neurofibrillary tangles. Significantly associated SNVs were identified. When results were meta-analyzed, *TMEM106B, GRN,* and *APOE* had significant gene-based associations with both LATE and HS, whereas *ABCC9* had significant associations with HS only. In a sensitivity analysis limited to LATE-NC + cases, *ABCC9* variants were again associated with HS. By contrast, the associations of *TMEM106B, GRN,* and *APOE* with HS were attenuated when adjusting for TDP-43 proteinopathy, indicating that these genes may be associated primarily with TDP-43 proteinopathy. None of these genes except *APOE* appeared to be associated with Alzheimer’s-type pathology. In summary, using data not included in prior studies of LATE or HS genomics, we replicated several previously reported gene-based associations and found novel evidence that specific risk alleles can differentially affect LATE-NC and HS.

**Supplementary Information:**

The online version contains supplementary material available at 10.1186/s40478-021-01250-2.

## Introduction

The present study focused on genetic contributions to transactive response DNA binding protein 43 kDa (TDP-43) proteinopathy and hippocampal sclerosis (HS). One or both of these pathologic features are observed in ~ 30% of brains among persons > 80 years at death [42]. The TDP-43 protein serves multiple functions in gene expression regulation at the levels of both transcription and translation [14, 21, 42, 52]. TDP-43 proteinopathy (aberrantly misfolded and mislocalized TDP-43 protein) is strongly associated with cognitive impairment [11, 41]. This pathologic hallmark was discovered in diseases that are now considered to be a clinical-pathologic spectrum that includes amyotrophic lateral sclerosis (ALS) and frontotemporal lobe degeneration with TDP-43 (FTLD-TDP) [50].

HS is a pathologic finding characterized by selective neuronal loss and gliosis of the hippocampal formation [3, 46]. First described as a pathologic phenomenon in epilepsy [19], HS is a descriptive and relatively nonspecific term used in both neuropathologic and neuroradiographic practice. However, in a subset of cases with HS, TDP-43 proteinopathy is also present [3, 11, 35, 46].

Limbic-predominant age-related TDP-43 encephalopathy (LATE) is a prevalent disease entity characterized by TDP-43 proteinopathy, with greatly increased risk for cognitive impairment, in aged populations [42]. LATE is not a subtype of FTLD-TDP because the associated disease(s) is not the frontotemporal dementia (FTD) clinical syndrome; rather, the presence of the neuropathologic changes underlying LATE (LATE-NC) is an amnestic dementia syndrome [40–42, 59]. HS pathology commonly co-occurs with LATE-NC and was the first neuropathologic change associated with the condition [17, 47]. However, some persons with LATE-NC have no HS, segmental/patchy HS, or unilateral HS [25]. It is currently unknown why some individuals with LATE-NC develop HS pathology while others do not, but genetics may help explain these phenomena.

Several genes and single nucleotide variants (SNVs) have been linked with LATE-NC phenotypes [42]. Risk for HS was previously associated with SNVs that are also known FTLD-TDP risk alleles, including rs5848 from the *GRN* gene on chromosome 17 and rs1990622 near the *TMEM106B* gene on chromosome 7 [6, 16, 39, 56, 62, 65]. In a genome-wide association study (GWAS), a SNV in the *ABCC9* gene (rs704178/rs704180) on chromosome 12 was associated with HS risk [43]. A separate GWAS found that rs9637454, an SNV in the *KCNMB2* gene on chromosome 3, was associated with HS risk [8]. Additional evidence exists linking the *APOE* ε4 allele, a strong risk factor for Alzheimer’s disease (AD), with increased HS and LATE-NC risk [60, 66, 68]. A study analyzing gene-based associations between the *GRN*, *TMEM106B*, *ABCC9*, and *KCNMB2* genes and HS found Bonferroni-corrected significant associations for *ABCC9* assuming a recessive mode of inheritance (MOI) and nominally significant associations with *GRN*, *TMEM106B*, and *KCNMB2* [29]. However, a separate study replicated the associations between *GRN* and *TMEM106B* SNVs with LATE-NC, but did not find an association between an *ABCC9* variant and LATE-NC or HS pathologies [23]. To the best of our knowledge, there has not been a prior study that found genomic associations with LATE-NC but not HS or vice versa.

In the current study, we analyzed genomic data from the Alzheimer’s Disease Genetics Consortium (ADGC) along with clinical and pathological data from the National Alzheimer’s Coordinating Center (NACC) and the Rush University Religious Orders Study and Memory and Aging Project (ROSMAP) to investigate the associations between prior identified putative risk genes – *KCNMB2*, *TMEM106B*, *ABCC9*, *GRN*, and *APOE* – and LATE-NC. While only analyzing participants not included in our prior studies [29, 43], we sought to test whether or not previously reported LATE-NC risk genes can be replicated for LATE-NC neuropathologic phenotypes (specifically, TDP-43 proteinopathy and HS) while also testing for the presence of novel risk alleles in those genes.

## Material and methods

### Study participants

Representative photomicrographs were taken, showing results from research participants with LATE-NC and LATE-NC + HS, in the University of Kentucky AD Research Center Autopsy cohort, using methods as previously described [44].

Phenotypic data from NACC (March 2021 data freeze) were linked with genotype data from the ADGC. Individuals who died at age 65 years or older were included. Similar to other studies using NACC data [27], individuals were excluded from the NACC cohort if at least one of 19 rare brain diseases were diagnosed (See Additional File 1: Supplemental Table 1) or if they were missing any adjustment variables or both endophenotypes under study.

The ROSMAP study has been described in detail elsewhere [36]. Briefly, data were acquired from two well-characterized cohort studies of aging and dementia. The Religious Orders Study (ROS), begun in 1994, and the Rush Memory and Aging Project (MAP), begun in 1997, involve older adults who enrolled without dementia, agreed to annual clinical evaluations and organ donation at death, and signed an Anatomical Gift Act for brain donation. Written informed consent was obtained from participants, and research was carried out in accordance with Institutional Review Board (IRB)-approved protocols. ROSMAP data are available online at the Rush Alzheimer’s Disease Center Resource Sharing Hub (https://www.radc.rush.edu/), as well as on the Accelerating Medicines Partnership-Alzheimer’s Disease (AMP-AD) Knowledge Portal (syn3219045).

For both the NACC and ROSMAP datasets, individuals were excluded from the analyses if they were included in either of two previous studies of HS genomics [29, 43]. In ROSMAP, participants were excluded based on IID if they were included in the Nelson et al. HS GWAS from 2014. In NACC, HS and TDP-43 were defined using variables from the v10 NACC Neuropathology (NP) dataset which were not available for the participants included in the previous studies. Thus, the NACC and ROSMAP participants included in the current study are a true replication cohort for these earlier HS genomics studies.

### Neuropathological endophenotype definitions

In the NACC NP dataset, LATE-NC was defined as either present or absent using the “distribution of TDP-43 immunoreactive inclusions” variables indicating if TDP-43 proteinopathy was observed in either the hippocampus (NPTDPC NACC field), entorhinal/inferior temporal cortex (NPTDPD), or neocortex (NPTDPE) in a case lacking overall diagnosis of FTLD-TDP. A LATE-NC case was defined as definitely having TDP-43 in the hippocampus, entorhinal/inferior temporal cortex, or neocortex. LATE-NC was considered unknown if TDP-43 data were unavailable in all three regions. HS was defined as either present or absent based on the “hippocampal sclerosis of CA1 and/or subiculum” (NPHIPSCL) variable using the “unilateral,” “bilateral,” and “present but laterality not assessed” response categories.

In the ROSMAP data set, LATE-NC was defined dichotomously using the “TDP-43 stage” (tdp_st4) variable and collapsing the 2nd and 3rd stages in cases lacking FTLD-TDP. HS was defined dichotomously by the “hippocampal sclerosis was rated as definitely present with CA1 region affected” response category of the “definite presence of typical hippocampal sclerosis” (hspath_typ) variable.

### Quality control of genotype data

For NACC participants, genomic data from the ADGC imputed using the Haplotype Reference Consortium (ADGC-HRC) were used [38]. The genetic data for ROSMAP were also imputed using the HRC and the methods have been described in detail elsewhere [18]. Standard GWAS quality control (QC) procedures were performed separately on the ADGC and ROSMAP genotype data using PLINK1.9 [37, 54]. SNVs were excluded if they were missing in more than 5% of samples, if they had a minor allele frequency less than 1%, or if they had Hardy–Weinberg Equilibrium (HWE) p-values < 1 × 10^–6^ among AD controls. Individuals were excluded if they were missing more than 5% of genotypes. Two individuals were considered related if they had an identity by descent measure of at least 0.25, which indicates that they are second-degree relatives. For related pairs, the individual with the lowest call rate was excluded.

NACC and ROSMAP genotype data were separately merged with 1000 Genomes Project Phase 3 data. Principal components (PCs) were calculated for the merged data sets using the “pca” procedure in PLINK1.9, and the first two PCs were plotted. The ADGC-HRC and ROSMAP individuals with first and second PCs that overlapped with those of the 1000 Genomes individuals of known European ancestry were identified and all other individuals were excluded from the analysis.

### Variant-level associations

All statistical analyses were conducted in R programming language [55], version 4.0.4. Associations between each endophenotype and each SNV were conducted separately in the NACC and ROSMAP datasets using binary logistic regression models assuming each of the three most common MOI: additive, dominant, and recessive. SNVs were excluded from the analyses if they were multiallelic or if there were fewer than 15 minor alleles present across all participants. All regression models were fit using the glm function in R assuming a binomial distribution and a logit link function and were adjusted for age at death, sex, ADGC data selection round (for NACC data) or ROS/MAP study (for ROSMAP data), and the first three genetic PCs. Odds ratios (OR) were calculated for each SNV. Since some endophenotypes were only available in a subset of participants, PCs were calculated separately for each endophenotype. NACC and ROSMAP SNV-level results were meta-analyzed using a fixed-effect, inverse-variance meta-analysis via the metagen function from the meta R package, version 4.18-0 [7]. For targeted analyses of previously reported SNVs, an additive MOI was assumed unless there existed previous evidence of association with another MOI. Additionally, LATE-NC-by-SNV interaction terms were tests for models of HS and were removed if they failed to reach statistical significance (p < 0.05). Plots of cohort-specific and meta-analyzed SNV-level p-values were created using LocusZoom Standalone, version 1.4 (https://genome.sph.umich.edu/wiki/LocusZoom_Standalone) [53], and the ggplot2 R package, version 3.3.3 [67]. Linkage disequilibrium estimates were computed using LDlink with the CEU population (https://ldlink.nci.nih.gov/) [34]. Jaccard similarity coefficients were used to estimate the similarity between binary variables and were calculated by dividing the size of their intersection by the size of their union via the clusteval R package, version 0.1 [58].

### Gene-based associations

Gene boundaries for *KCNMB2*, *TMEM106B*, *ABCC9*, *GRN*, and *APOE* were defined based on their canonical transcripts using the Genome Reference Consortium Human Build 37 (GRCh37/hg19) gene range list from PLINK (https://www.cog-genomics.org/plink/1.9/resources). All genes were flanked by an additional 10 kb to include potential regulatory regions. See Additional File 1: Supplemental Table 2 for the positions used to define the gene boundaries.

For each gene, endophenotype, and MOI, all SNV-level p-values were combined using the aggregated Cauchy association test (ACAT) [33]. All ACAT analyses were run using R functions provided by the authors (https://github.com/yaowuliu/ACAT). Equal weights were assumed for all SNVs in the ACAT analyses and statistical significance was defined as a p-value < 0.05.

### SNV prioritization and follow-up analyses

Prioritized SNVs were identified using a Bonferroni-corrected threshold for significance that accounts for the effective number of independent tests in a given genetic region. The effective number of independent tests in a region was calculated for each endophenotype using the method of Gao et al. [20]. Briefly, Pearson’s correlation coefficient was calculated for all pairs of SNVs and these coefficients were placed in a square matrix. The eigenvalues of the matrix were then computed and ordered from largest to smallest and the effective number of independent tests was defined to be the smallest number of ordered eigenvalues that account for 99.5% of the sum of all eigenvalues. The Bonferroni-corrected threshold for identifying prioritized SNVs in a given genetic region was defined as 0.05 divided by the largest estimated number of independent tests in the region.

Prioritized SNVs were investigated for expression quantitative trait loci (eQTL) associations using the Genotype-Tissue Expression (GTEx) Project’s V8 public data [15], the BRAINEAC Brain eQTL Almanac (http://braineac.org/) [57], and Functional Annotation of Human Long Noncoding RNAs via Molecular Mapping (FANTOM5) database (data accessed via: https://www.ebi.ac.uk/gxa/experiments/E-MTAB-3358/Results). Prioritized SNVs were also investigated for associations with other molecular mechanisms using the INFERring the molecular mechanisms of NOncoding genetic variants (INFERNO) software assuming a threshold on r^2^ of 0.5 and a threshold on LD block size of 500 kb (http://inferno.lisanwanglab.org/index.php) [4].

### Sensitivity analyses

Additional analyses tested if the study’s results were dependent upon a priori analytic approaches. All gene-based analyses were also conducted assuming 0 kb and 25 kb of flanking around each gene. All top SNVs were tested for associations with AD-related neuropathologies, to see if there were indications that the HS and LATE-NC associations were being driven by AD. Additionally, since TDP-43 in the amygdala was not included in the dichotomous LATE-NC definition, all top SNVs were also tested for associations with LATE-NC Stage 1 (vs. LATE-NC Stage 0) to determine if any amygdala-specific associations were missed in the primary analyses.

## Results

**Fig. 1:**
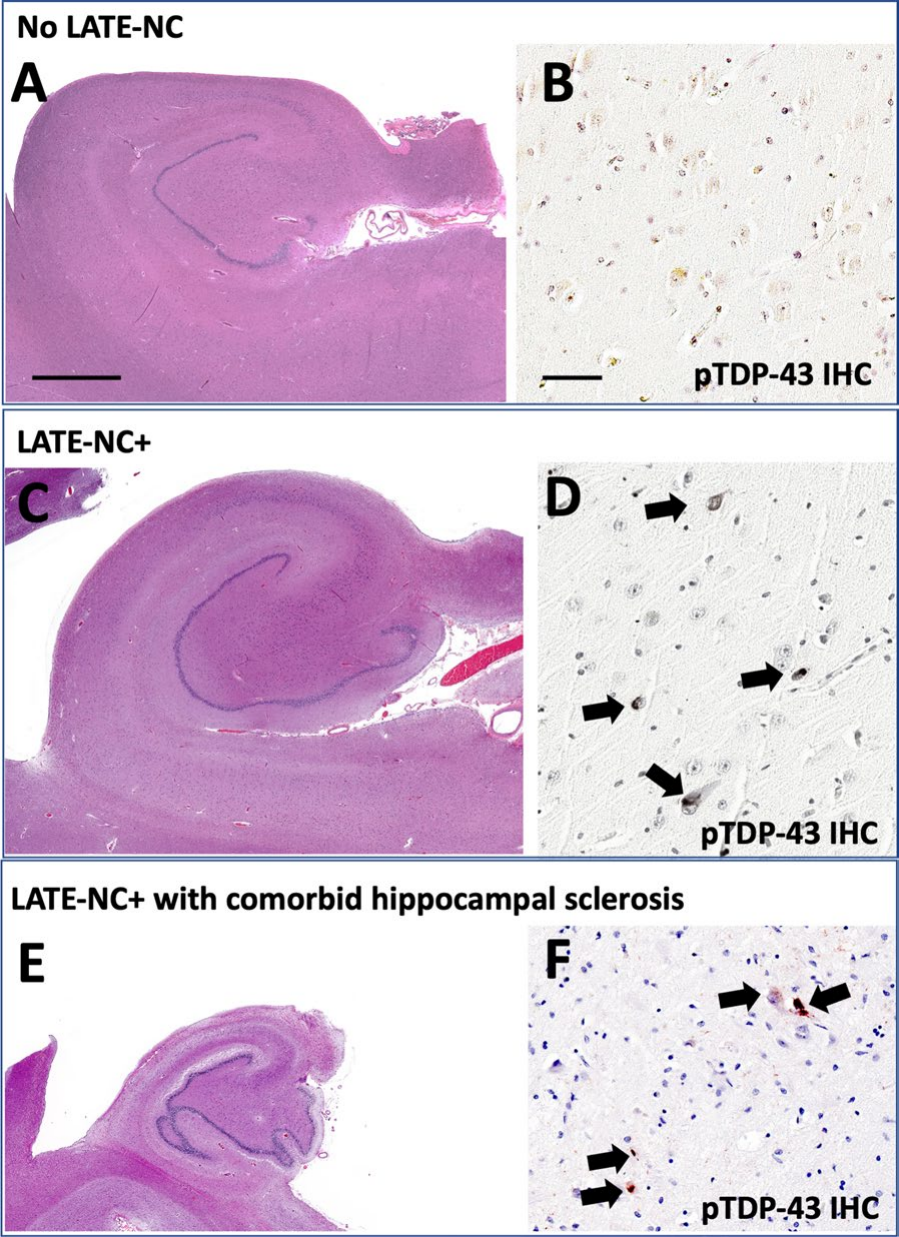
Photomicrographs of human hippocampi depict the main neuropathologic endophenotypes analyzed in the current study. Hippocampal sclerosis (HS) is evaluated with H&E stain (panels **A**, **C**, **E**), whereas LATE-NC is operationalized with phospho-TDP-43 immunohistochemistry (IHC; panels **B**, **D**, and **F**). All photomicrographs depict mid-level hippocampal sections dissected in the coronal plane. Panels **A** and **B** show stained brain sections from a woman (APOE e3/e4) who died at age 83; autopsy revealed neither LATE-NC nor HS. Panels **C** and **D** are from a man (APOE e3/e4) who died at age 93 with LATE-NC Stage 2. Panels **E** and **F** are from a woman (APOE e3/e3) who died at age 95 with LATE-NC Stage 2 and comorbid HS. Note the relatively atrophic hippocampal profile in Panel **E** in comparison to** a** or **c** (same scale bar); the HS + profile in panel **E** also demonstrates parenchymal rarefaction which can be appreciated even at low magnification. Phospho-TDP-43 immunoreactive intraneuronal inclusions are highlighted with arrows in panels **D** and **F**. The representative photomicrographs were from research participants of the University of Kentucky AD Research Center. Scale bar = 2 mm in **A**, **C**, and **E**, 75 microns in **A**, **D**, and **F**

The phenotypes of interest in the current study are autopsy-confirmed LATE-NC and HS. Specific examples of those pathologies are depicted in Fig. 1. Some brains have LATE-NC without HS (Fig. 1b). However, individuals with LATE-NC are at increased risk of having comorbid HS (Fig. 1c).

**Fig. 2:**
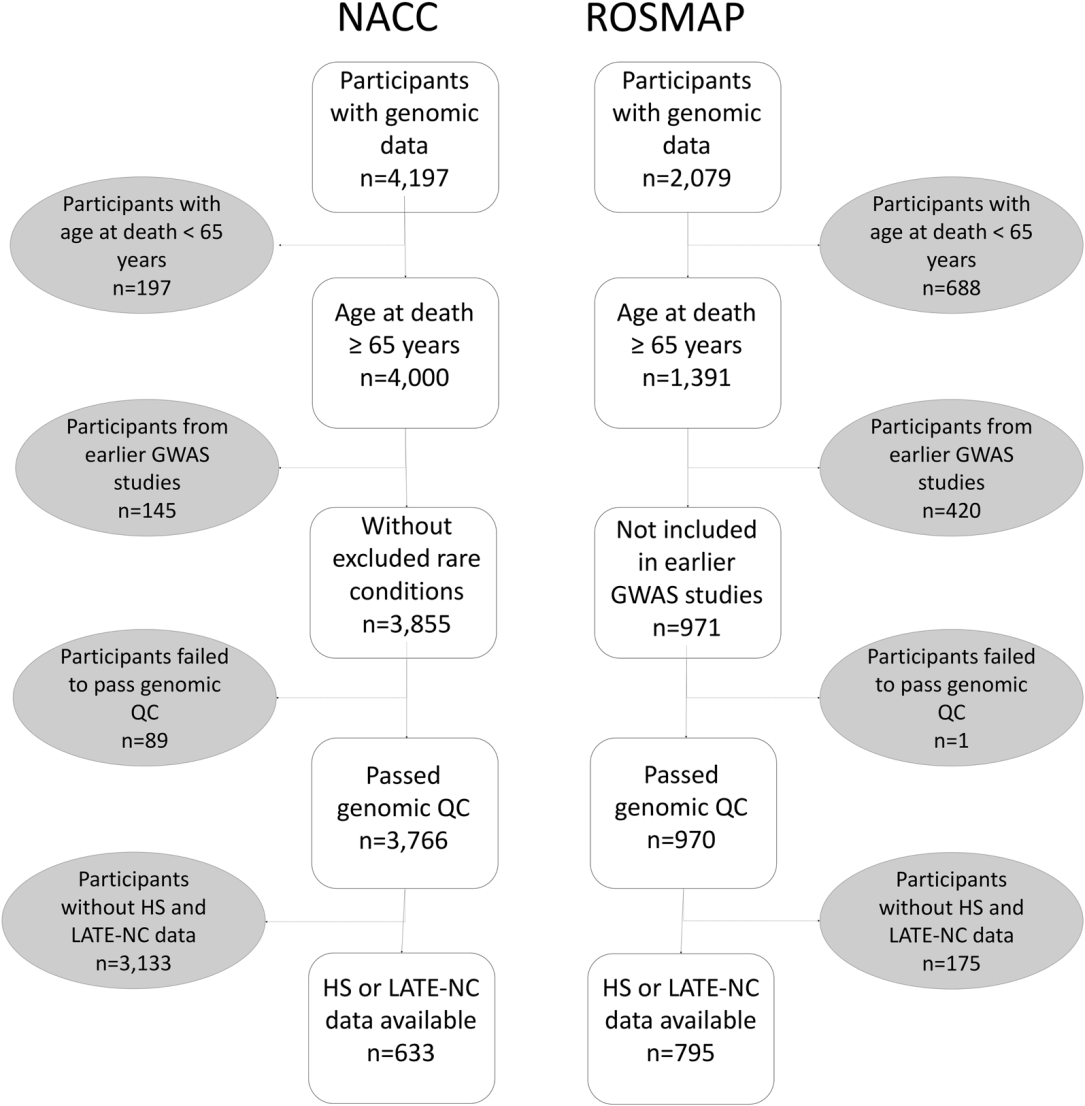
Included and excluded research participants, along with criteria for exclusion. A flowchart summarizing inclusions and exclusions for National Alzheimer's Coordinating Center (NACC) and Religious Orders Study and Rush Memory and Aging Project (ROSMAP) participants

The participants included and excluded, and the reasons for exclusion, are shown in Fig. 2. In the ROSMAP data set, a total of n = 795 individuals had available data for at least one of the endophenotypes along with GWAS data and were not included in earlier studies of HS [29, 43]. In the NACC data set, n = 633 individuals had available data for at least one of the endophenotypes along with GWAS data and were not included in the earlier studies of HS [29, 43]. While not all FTLD subtypes were explicitly excluded among NACC participants, no FTLD cases were included in the final sample likely due inclusion criteria applied by the ADGC during genotyping. Table 1 shows a summary of individual characteristics and endophenotypes for both NACC and ROSMAP participants. ROSMAP participants tended to be older at death (p < 0.001), were more likely to be female (p < 0.001), and were less likely to be an HS case (p = 0.007) than NACC participants. HS was less prevalent than LATE-NC in both cohorts (NACC: HS 14.1%, LATE-NC 29.4%; ROSMAP: HS 9.4%, LATE-NC 33.2%). In ROSMAP, both HS cases and LATE-NC cases tended to be older at death (both p < 0.001) and were less likely to be male (p = 0.054 and p < 0.001, respectively) than their respective controls. There were no identified statistically significant differences in basic demographic characteristics between HS and/or LATE-NC cases and their respective controls in NACC. Additional participant characteristics stratified by combined LATE-NC and HS case status are included in Additional File 1: Supplemental Tables 4 and 5.

**Table 1:** Participant characteristics stratified by endophenotype status

	NACC			ROSMAP		
	Number of Participants (%)	Age at Death, Mean (SD)	Female, N (%)	Number of Participants (%)	Age at Death, Mean (SD)	Female, N (%)
HS						
Overall	N = 631	85.9 (8.3)	319 (50.6)	N = 780	88.7 (7.2)	525 (67.3)
No	542 (85.9)	85.9 (8.4)	270 (49.8)	707 (90.6)	88.3 (7.2)	468 (66.2)
Yes	89 (14.1)	86.0 (7.5)	49 (55.1)	73 (9.4)	92.0 (6.4)	57 (78.1)
LATE-NC						
Overall	N = 512	85.1 (7.9)	207 (50.2)	N = 747	89.1 (7.1)	506 (67.7)
No	291 (70.6)	84.9 (8.1)	138 (47.4)	499 (66.8)	87.9 (7.3)	315 (63.1)
Yes	121 (29.4)	85.4 (7.3)	66 (57.0)	248 (33.2)	91.5 (6.1)	191 (77.0)

Participant characteristics stratified by hippocampal sclerosis (HS) and limbic-predominant age-related TDP-43 encephalopathy neuropathological changes (LATE-NC) case status. NACC = National Alzheimer's Coordinating Center; ROSMAP = Religious Orders Study and Rush Memory and Aging Project; SD = standard deviation; HS = hippocampal sclerosis; LATE-NC = limbic-predominant age-related TDP-43 encephalopathy neuropathological changes

**Fig. 3:**
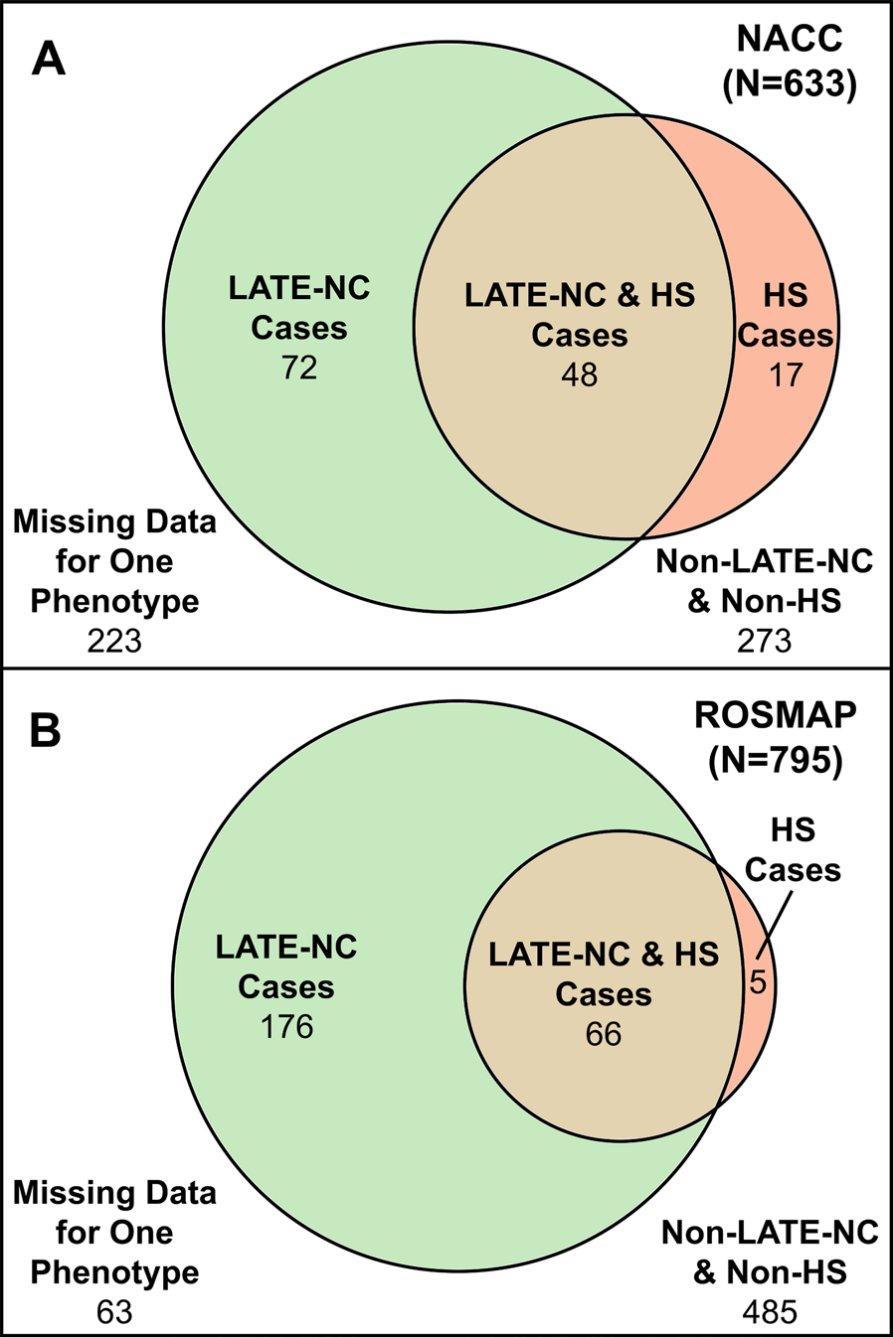
Venn diagrams of the overlap between endophenotypes across studies. Venn diagrams of the overlap between limbic-predominant age-related TDP-43 encephalopathy neuropathological change (LATE-NC) and hippocampal sclerosis (HS) cases in **A.** National Alzheimer's Coordinating Center (NACC) and **B.** Religious Orders Study and Rush Memory and Aging Project (ROSMAP). LATE-NC = limbic-predominant age-related TDP-43 encephalopathy neuropathological change; HS = hippocampal sclerosis; NACC = National Alzheimer's Coordinating Center; ROSMAP = Religious Orders Study and Rush Memory and Aging Project

Persons with HS tended to also have LATE-NC and the reverse was also true among individuals in both datasets (Jaccard coefficients of 0.589 and 0.575 in NACC and ROSMAP, respectively); see Fig. 3. Of the 732 ROSMAP participants with available case data for both LATE-NC and HS, 93% of HS cases were also LATE-NC cases. Of the 410 NACC participants with available case data for both LATE-NC and HS, 73% of HS cases were also LATE-NC cases.

Across the *KCNMB2*, *TMEM106B*, *ABCC9*, *GRN*, and *APOE* genes, each flanked by 10 kb, a total of 1,580 SNVs passed QC in NACC while 1,532 SNVs passed QC in ROSMAP. A total of 1,438 SNVs were shared between NACC and ROSMAP and were included in the meta-analysis (Additional File 1: Supplemental Table 2).

### Gene-based associations

The adjusted meta-analyzed, SNV-level results were combined within genes via ACAT to obtain gene-based p-values. At the gene level, *TMEM106B* and *APOE* were significantly associated with both HS and LATE-NC while *ABCC9* and *GRN* were significantly associated with HS only (Table 2). Neither HS nor LATE-NC were significantly associated with *KCNMB2*. The meta-analyzed gene-based results were largely similar to when they were conducted separately in the NACC and ROSMAP datasets. Additionally, these results were largely unchanged when 0 kb and 25 kb of flanking were added to each gene.

**Table 2:** Gene-based results for risk genes

Chr	Gene	Endophenotype	MOI		
			Additive	Dominant	Recessive
3	*KCNMB2*	HS	0.718	0.632	0.478
		LATE-NC	0.980	0.995	0.473
7	*TMEM106B*	HS	**0.006**	0.052	**0.005**
		LATE-NC	** < 0.001**	**0.004**	** < 0.001**
12	*ABCC9*	HS	**0.036**	0.072	**0.006**
		LATE-NC	0.901	0.440	0.912
17	*GRN*	HS	**0.004**	0.348	**0.003**
		LATE-NC	0.164	0.628	0.069
19	*APOE*	HS	**0.014**	**0.017**	0.333
		LATE-NC	** < 0.001**	** < 0.001**	0.064

Aggregated Cauchy association test (ACAT) gene-based p-values for hippocampal sclerosis (HS) and limbic-predominant age-related TDP-43 encephalopathy neuropathological changes (LATE-NC). Each gene is flanked by 10 kb. All SNV-level analyses were adjusted for sex, age at death, cohort/study, and the first three genetic principal components and meta-analyzed across National Alzheimer's Coordinating Center (NACC) and Religious Orders Study and Rush Memory and Aging Project (ROSMAP) participants. Chr. = chromosome; HS = hippocampal sclerosis; LATE-NC = limbic-predominant age-related TDP-43 encephalopathy neuropathological changes; MOI = mode of inheritance.

### Prioritized SNVs and follow-up analyses

The effective number of independent tests for *TMEM106B* ± 10 kb was estimated to be 25, *GRN* ± 10 kb was estimated to be 16, *KCNMB2* ± 10 kb was estimated to be 104, *APOE* ± 10 kb was estimated to be 14, and *ABCC9* ± 10 kb was estimated to be 71. The Bonferroni-corrected thresholds for a genetic region was calculated by dividing 0.05 by the corresponding estimated effective number of independent tests in the region.

**Fig. 4:**
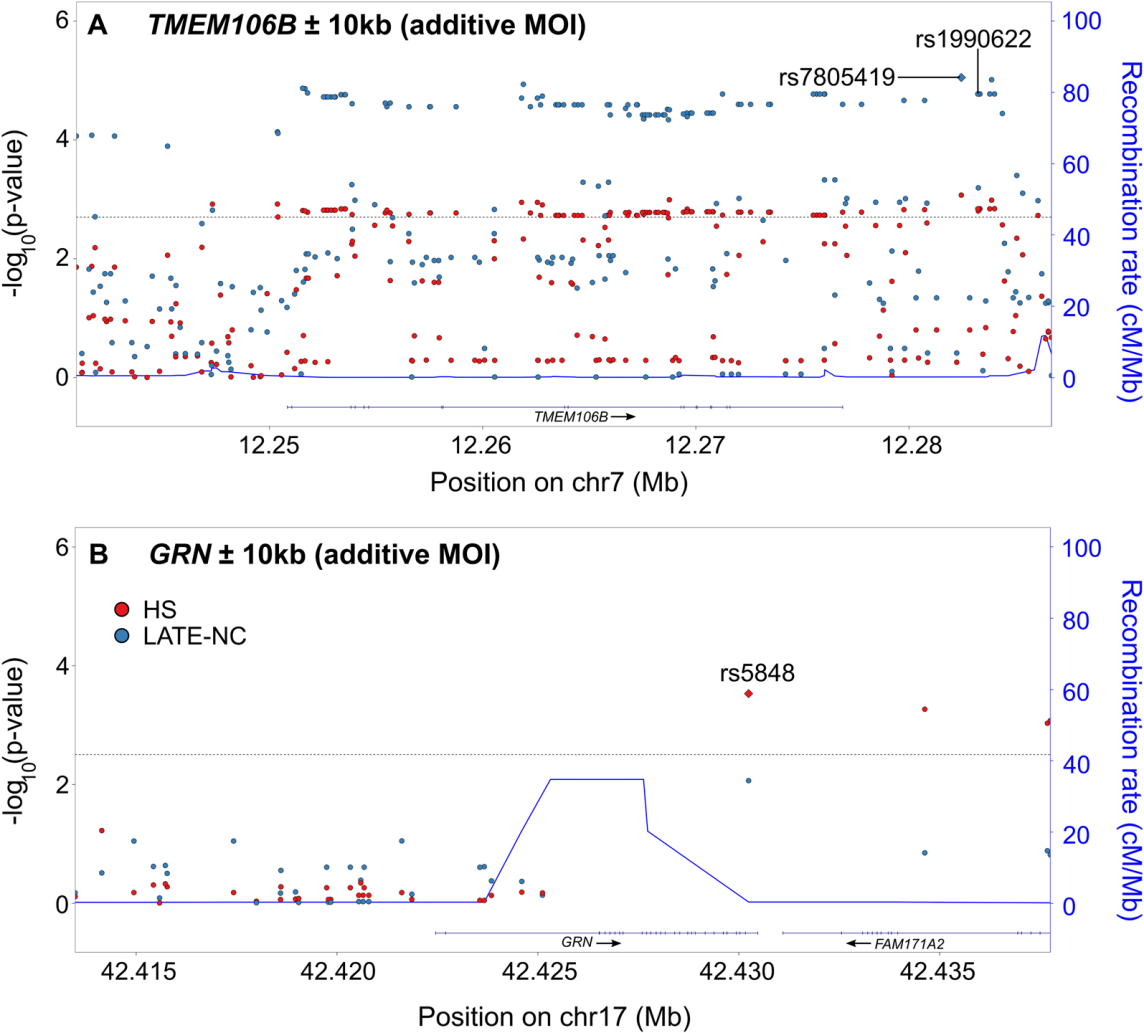
Variant-level results for *TMEM106B* and *GRN*. Adjusted, meta-analytic, single nucleotide variant (SNV)-level p-values for hippocampal sclerosis (HS) and limbic-predominant age-related TDP-43 encephalopathy neuropathological change (LATE-NC) across **A.** *TMEM106B* ± 10 kb and **B.** *GRN* ± 10 kb. All analyses were adjusted for sex, age at death, cohort/study, and the first three genetic principal components. Horizontal dashed lines represent the Bonferroni-corrected thresholds for significance that account for the number of independent tests in each genomic region. A diamond represents the SNV with the smallest p-value. The previously identified *TMEM106B* SNV (Rutherford et al. [62]) is labeled and identified with an arrow. MOI = mode of inheritance; LATE-NC = limbic-predominant age-related TDP-43 encephalopathy neuropathological change; HS = hippocampal sclerosis

One hundred and ten SNVs in the *TMEM106B* ± 10 kb locus had adjusted meta-analytic associations with HS or LATE-NC less than the Bonferroni-corrected threshold (Fig. 4a). At the *TMEM106B* ± 10 kb locus, rs7781670 had the smallest adjusted meta-analytic p-value for LATE-NC assuming an additive MOI (p = 2.97 × 10^–5^). rs7781670 also met the Bonferroni-corrected threshold for the *TMEM106B* ± 10 kb locus for HS when assuming a recessive MOI (p = 1.63 × 10^–3^) and was a significant eQTL in GTEx for *TMEM106B* in the cerebellum (p = 4.7 × 10^–7^) and the cortex (p = 2.6 × 10^–5^). In INFERNO, these prioritized SNVs were associated with both eQTLs and Roadmap enhancers in blood, connective, and epithelial tissues and just Roadmap enhancers in brain, heart, immune organ, liver, and skeletal tissues, among others.

Fourteen SNVs in the *GRN* ± 10 kb locus had adjusted meta-analytic associations with HS or LATE-NC less than the Bonferroni-corrected threshold (Fig. 4b). rs5848 had the smallest adjusted meta-analytic p-value in the *GRN* ± 10 kb locus and met the Bonferroni-corrected threshold for HS (additive MOI p = 2.16 × 10^–4^; recessive MOI p = 1.91 × 10^–4^). rs5848 also had the smallest adjusted meta-analytic p-value for LATE-NC in the *GRN* ± 10 kb locus, but it did not meet the Bonferroni-corrected threshold. In GTEx, rs5848 was a significant eQTL for *GRN* expression in numerous tissues including thyroid (p = 2.2 × 10^–16^), caudate (p = 2.0 × 10^–12^), cortex (p = 2.0 × 10^–9^), and frontal cortex (p = 4.4 × 10^–9^). In INFERNO, these prioritized SNVs were associated with both eQTLs and Roadmap enhancers in adipose, connective, endocrine, heart, and nervous tissues, with just eQTLs in blood vessel tissue, and with just Roadmap enhancers in brain, blood, immune organ, liver, and skeletal muscle tissues, among others.

No SNVs in the *KCNMB2* ± 10 kb locus had adjusted meta-analytic associations with HS or LATE-NC that met the Bonferroni-corrected threshold (Additional File 1: Supplemental Fig. 1).

**Fig. 5:**
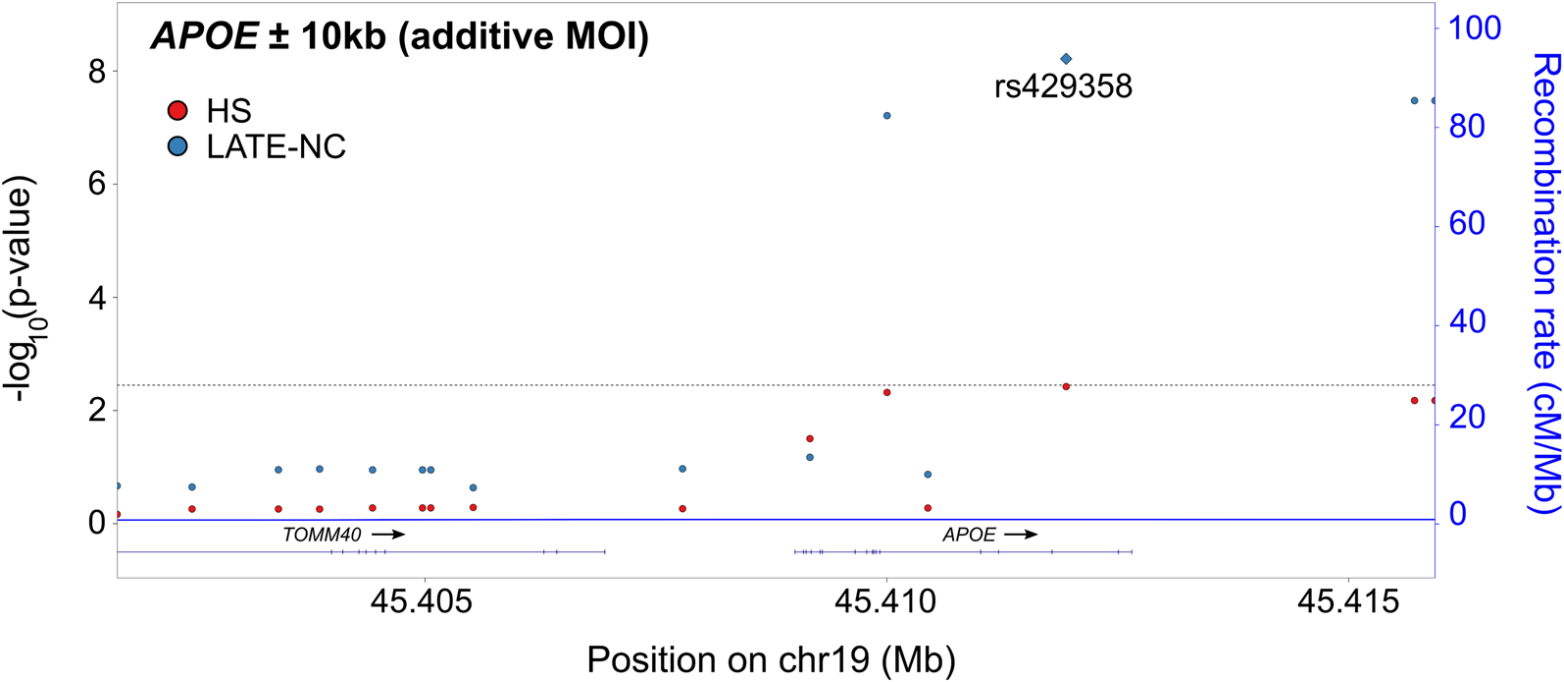
Variant-level results for *APOE*. Adjusted, meta-analytic, single nucleotide variant (SNV)-level p-values for hippocampal sclerosis (HS) and limbic-predominant age-related TDP-43 encephalopathy neuropathological change (LATE-NC) across *APOE* ± 10 kb. All analyses were adjusted for sex, age at death, cohort/study, and the first three genetic principal components. The horizontal dashed line represents the Bonferroni-corrected threshold for significance that accounts for the number of independent tests in the *APOE* ± 10 kb region. A diamond represents the SNV with the smallest p-value. MOI = mode of inheritance; LATE-NC = limbic-predominant age-related TDP-43 encephalopathy neuropathological change; HS = hippocampal sclerosis

The *APOE* ± 10 kb locus was strongly associated with LATE-NC. Four SNVs (rs429358, rs769449, rs10414043, and rs7256200), all in high linkage disequilibrium with one another (all r^2^ > 0.95), had adjusted meta-analytic associations with LATE-NC that met the Bonferroni-corrected threshold assuming an additive MOI (all p-values ≤ 2.56 × 10^–8^) (Fig. 5). While none of the *APOE* SNVs were associated with *APOE* expression levels in the evaluated data sets, rs769449 and rs10414043 were significant sQTLs in GTEx for *TOMM40* in cerebellar hemisphere tissue (p = 4.0 × 10^–10^ and p = 1.4 × 10^–5^, respectively). In INFERNO, these prioritized SNVs were associated with both Roadmap and FANTOM5 enhancers in adipose, blood, brain, connective, epithelial, liver, nervous, skeletal muscle, smooth muscle, and stem cell tissues and with just Roadmap enhancers in endocrine, heart, and immune organ tissues, among others.

**Fig. 6:**
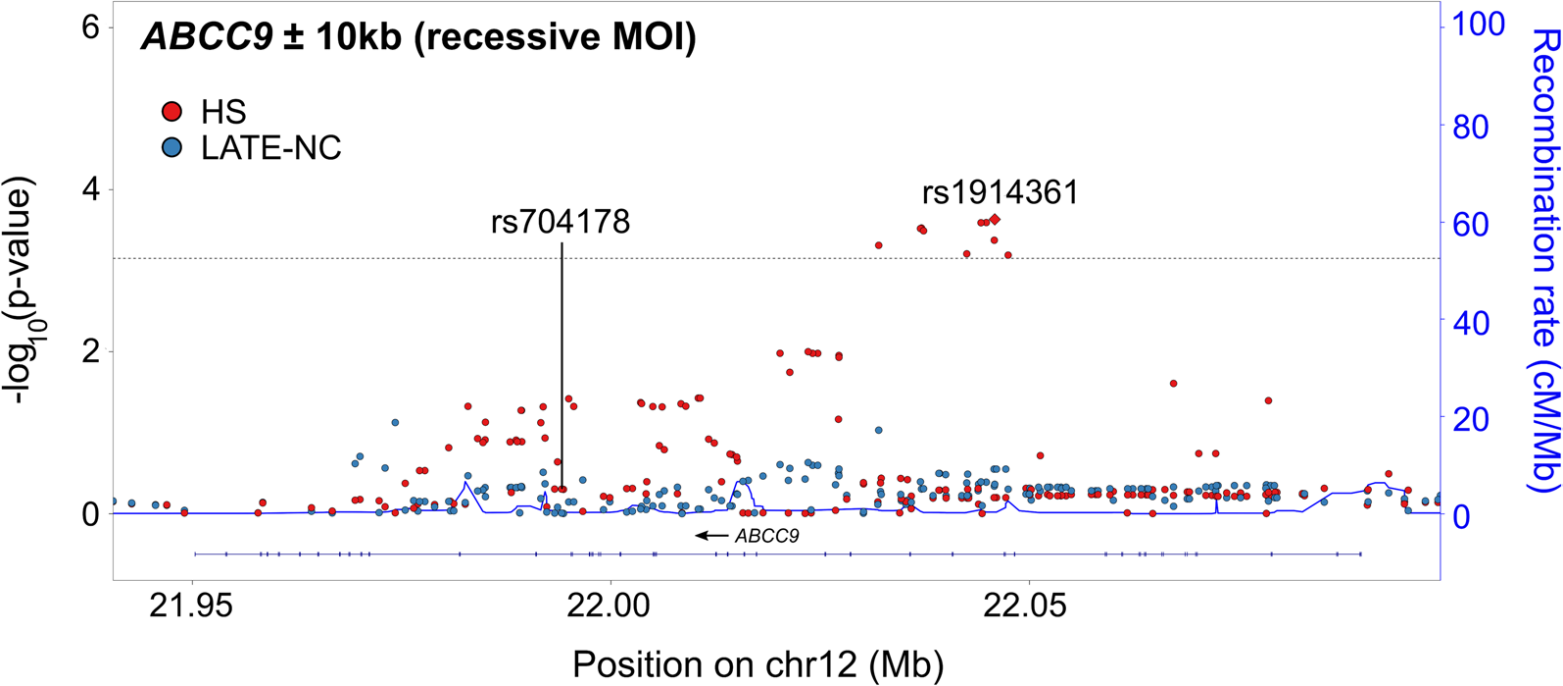
Variant-level results for *ABCC9*. Adjusted, meta-analytic, single nucleotide variant (SNV)-level p-values for hippocampal sclerosis (HS) and limbic-predominant age-related TDP-43 encephalopathy neuropathological change (LATE-NC) across *ABCC9* ± 10 kb assuming a recessive mode of inheritance (MOI). A recessive MOI was assumed for *ABCC9* since it has consistently been the MOI with the strongest HS association for *ABCC9* [43, 48, 29]. All analyses were for sex, age at death, cohort/study, and the first three genetic principal components. The horizontal dashed line represents the Bonferroni-corrected threshold for significance that accounts for the number of independent tests in the *ABCC9* ± 10 kb region. A diamond represents the SNV with the smallest p-value. The previously identified *ABCC9* SNV [43] is labeled and identified with an arrow. MOI = mode of inheritance; LATE-NC = limbic-predominant age-related TDP-43 encephalopathy neuropathological change; HS = hippocampal sclerosis

**Fig. 7:**
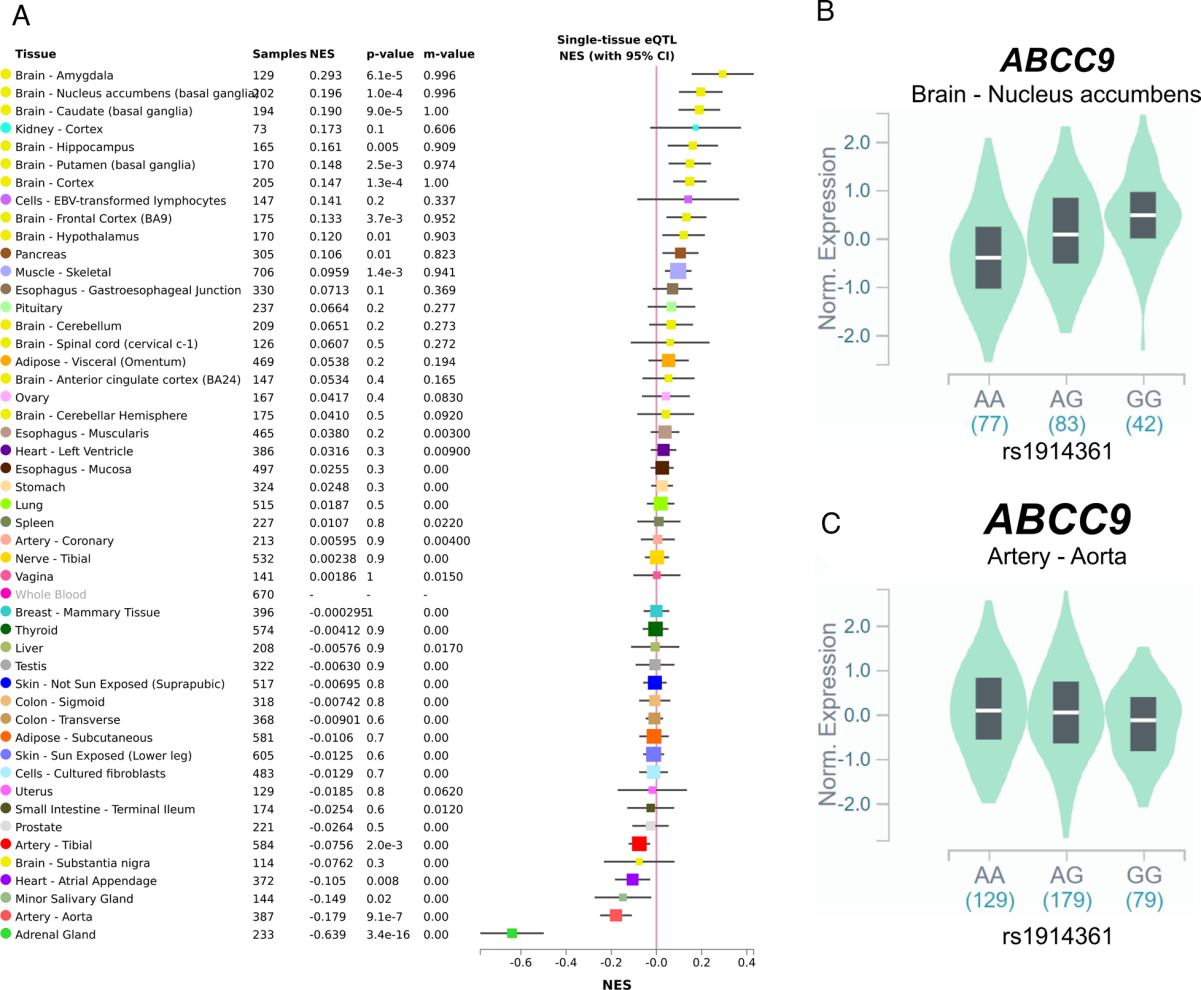
Expression quantitative trait loci (eQTL) analyses for rs1914361 and *ABCC9* across tissue types. Expression quantitative trait loci (eQTL) analyses for rs1914361 and *ABCC9* gene expression across human tissues in the Genotype-Tissue Expression (GTEx) database. **a** Multi-tissue eQTL plot of rs1914361 and *ABCC9* gene expression; **b** *ABCC9* normalized gene expression stratified by rs1914361 minor alleles in the nucleus acumbens region of the brain; and **c** *ABCC9* normalized gene expression stratified by rs1914361 minor alleles in the aorta region of the artery. GTEx = Genotype-Tissue Expression; NES = normalize effect size; eQTL = expression quantitative trait loci

The *ABCC9* ± 10 kb locus was most strongly associated with HS and contained 13 SNVs with adjusted meta-analytic p-values for HS less than the Bonferroni-corrected threshold (Fig. 6). rs1914361 had the smallest adjusted meta-analytic p-value with HS assuming a recessive MOI (p = 1.70 × 10^–4^). In prior studies with cohorts of research subjects that did not overlap with the current study, the *ABCC9*/HS association was strongest for the recessive MOI models [29, 43, 48]. All other SNVs that also met the Bonferroni-corrected threshold when assuming a recessive MOI were in high linkage disequilibrium with rs1914361 (all r^2^ > 0.75). rs1914361 was a significant eQTL in the GTEx data set for the expression of *ABCC9* in several tissues, including brain (nucleus accumbens, caudate, cortex, and putamen) and artery tissues (tibial and aorta) (Fig. 7a). Notably, rs1914361 minor alleles were positively correlated with *ABCC9* expression in brain tissues (Fig. 7b) and negatively correlated with *ABCC9* expression in artery tissues (Fig. 7c). Furthermore, relative to rs704178, a previously identified *ABCC9* HS SNV, rs1914361 had a similarly strong association with *ABCC9* gene expression in GTEx (rs704178: p = 4.00 × 10^–13^; rs1914361: p = 7.10 × 10^–12^) and a stronger association with *ABCC9* gene expression in BRAINEAC (rs704178: p = 6.80 × 10^–4^; rs1914361: p = 2.10 × 10^–7^) (Table 3). In INFERNO, these prioritized SNVs were associated with Roadmap enhancers in adipose, blood vessel, connective, heart, live, skeletal muscle, and smooth muscle tissues, among others.

**Table 3:** Most significant expression quantitative trait loci (eQTL) p-values for *ABCC9* in BRAINEAC and GTEx databases

Gene	SNV	Most Significant eQTL P-value	
		BRAINEAC	GTEx
*ABCC9*	rs704178	6.80E-04	4.00E-13
	rs1914361	2.10E-07	7.10E-12

eQTL = expression quantitative trait loci; GTEx = Genotype-Tissue Expression; SNV = single-nucleotide variant

### SNV-level regression analyses

In their respective regression models, the *GRN* SNV rs5848 (p = 0.010), the *APOE* SNV rs769449 (p < 0.001), and *APOE* ε4 carrier status (p < 0.001) all had nominally significant adjusted meta-analytic associations with LATE-NC and the *TMEM106B* SNV rs7781670 had a borderline-significant adjusted meta-analytic association with LATE-NC (p = 0.057) (Table 4). All odds ratio estimates were consistent across NACC and ROSMAP with the exception of the *ABCC9* SNV rs1914361 when assuming a recessive MOI (NACC: OR = 0.98; ROSMAP: OR = 1.40). Notably, the odds ratio estimates for LATE-NC were very similar between the *APOE* SNV rs769449 (meta-analytic OR = 1.95) and *APOE* ε4 carrier status (meta-analytic OR = 2.05), which likely reflects the fact that rs769449 minor allele counts are strongly correlated with *APOE* ε4 counts (NACC: r^2^ = 0.746; ROSMAP: r^2^ = 0.712). In a sensitivity analysis with smaller sample sizes, the *TMEM106B* SNV rs1990622, but none of the other tested SNVs, had nominally significant adjusted associations with TDP-43 in the amygdala (LATE-NC Stage 1 vs. LATE-NC Stage 0); see Additional File 1: Supplemental Table 6.

**Table 4:** Adjusted limbic predominant age-related TDP-43 encephalopathy neuropathological changes (LATE-NC) odds ratios for risk variants

Gene	MOI	SNV	Effect Allele	NACC		ROSMAP		Meta-Analysis		
				OR	P-value	OR	P-value	OR	95% CI	P-value
*TMEM106B*	Additive	rs1990622	A	1.39	0.051	1.08	0.484	1.16	(0.97, 1.39)	0.099
*TMEM106B*	Additive	rs7781670	C	1.47	**0.024**	1.09	0.415	1.19	(1.00, 1.43)	0.057
*GRN*	Additive	rs5848	T	1.40	**0.042**	1.23	0.089	1.29	(1.06, 1.56)	**0.010**
*ABCC9*	Additive	rs1914361	G	1.16	0.354	1.16	0.171	1.16	(0.97, 1.39)	0.098
*ABCC9*	Recessive	rs1914361	G	0.98	0.933	1.40	0.077	1.25	(0.92, 1.71)	0.151
*ABCC9*	Additive	rs704178	G	0.95	0.764	1.07	0.536	1.03	(0.86, 1.24)	0.732
*ABCC9*	Recessive	rs704178	G	0.80	0.433	1.16	0.394	1.05	(0.78, 1.40)	0.759
*APOE*	Additive	rs769449	A	1.70	**0.004**	2.22	**< 0.001**	1.95	(1.51, 2.52)	** < 0.001**
*APOE*	N/A	e4 Carrier	N/A	1.88	**0.010**	2.16	**< 0.001**	2.05	(1.54, 2.74)	**< 0.001**

Adjusted effects of single nucleotide variants (SNV) on limbic-predominant age-related TDP-43 encephalopathy neuropathological change (LATE-NC). All models adjust for sex, age at death, first three principal components and cohort/study. For rs1990622, rs7781670, and rs704178, the effect alleles are the risk-associated alleles and not the minor alleles. NACC = National Alzheimer's Coordinating Center; ROSMAP = Religious Orders Study and Rush Memory and Aging Project; MOI = mode of inheritance; SNV = single-nucleotide variant; OR = odds ratio; CI = confidence interval.

**Fig. 8:**
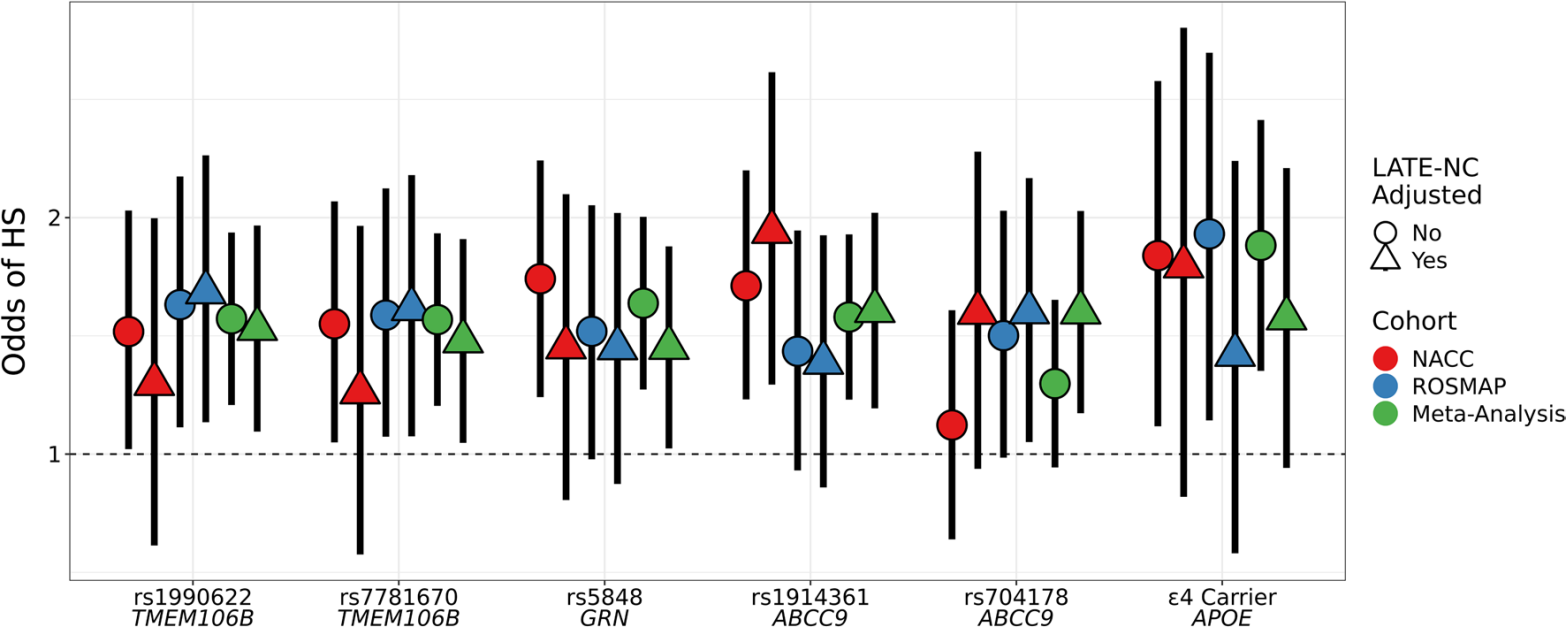
Adjusted odds ratios for hippocampal sclerosis (HS) across variants. Adjusted odds ratio estimates and 95% confidence intervals for genetic single nucleotide variants (SNV) and *APOE* ε4 carrier status from separate regression models of hippocampal sclerosis (HS) fit using data from the National Alzheimer's Coordinating Center (NACC), the Religious Orders Study and Rush Memory and Aging Project (ROSMAP), and the meta-analysis of NACC and ROSMAP. All regression models were adjusted for sex, age at death, cohort/study, and the first three genetic principal components. Regression models were also adjusted for limbic-predominant age-related TDP-43 encephalopathy neuropathological change (LATE-NC) case status by the including LATE-NC status as an additional predictor variable and these odds ratio estimates are represented by triangles. For each variant, the effect allele is defined as the HS risk-causing allele (HA odds ratio estimates > 1.0), and not necessarily the minor allele. An additive mode of inheritance (MOI) is assumed for all variants except for rs704178 where a dominant MOI was assumed (since a recessive MOI resulted in a significant protective effect for HS). HS = hippocampal sclerosis; LATE-NC = limbic-predominant age-related TDP-43 encephalopathy neuropathological change; NACC = National Alzheimer's Coordinating Center; ROSMAP = Religious Orders Study and Rush Memory and Aging Project

No LATE-NC-by-SNV interactions were significant in the adjusted HS models, so the interaction terms were removed. The *TMEM106B* SNVs (rs1990622 and rs7781670), the *GRN* SNV (rs5848), one of the *ABCC9* SNVs (rs1914361), the *APOE* SNV (rs769449), and *APOE* ε4 carrier status all had nominally significant, adjusted meta-analytic associations with HS (Table 5). When these models were adjusted for LATE-NC, all models had nominally significant adjusted meta-analytic associations with HS with the exception of the *APOE* SNV (rs769449) and *APOE* ε4 carrier status (Table 5, Fig. 8), suggesting that the association between *APOE* status and HS is related to a more direct interaction between *APOE* and LATE-NC (i.e., TDP-43 proteinopathy). By contrast, the association between HS and the *ABCC9* SNV rs704178 becomes nominally significant with larger odds ratio estimates when adjusted for LATE-NC.

**Table 5:** Hippocampal sclerosis (HS) odds ratios for risk variants with and without adjustment for LATE-NC status

Gene	MOI	SNV	Effect Allele	LATE-NC Adjusted	NACC		ROSMAP		Meta-Analysis		
					OR	P-value	OR	P-value	OR	95% CI	P-value
*TMEM106B*	Additive	rs1990622	A	Yes	1.23	0.396	1.61	**0.017**	1.44	(1.07, 1.95)	**0.017**
				No	1.43	**0.044**	1.55	**0.019**	1.49	(1.15, 1.91)	**0.002**
*TMEM106B*	Additive	rs7781670	C	Yes	1.20	0.456	1.53	**0.029**	1.39	(1.03, 1.88)	**0.030**
				No	1.47	**0.034**	1.50	**0.028**	1.48	(1.15, 1.91)	**0.002**
*GRN*	Additive	rs5848	T	Yes	1.37	0.168	1.37	0.123	1.37	(1.02, 1.84)	**0.039**
				No	1.67	**0.004**	1.43	0.057	1.56	(1.21, 2.00)	**< 0.001**
*ABCC9*	Additive	rs1914361	G	Yes	1.92	**0.005**	1.31	0.152	1.52	(1.14, 2.03)	**0.004**
				No	1.64	**0.004**	1.35	0.092	1.49	(1.17, 1.90)	**0.001**
*ABCC9*	Recessive	rs1914361	G	Yes	3.87	**< 0.001**	1.58	0.124	2.23	(1.42, 3.51)	**< 0.001**
				No	2.69	**< 0.001**	1.64	0.075	2.12	(1.45, 3.09)	**< 0.001**
*ABCC9*	Additive	rs704178	G	Yes	1.51	0.079	1.52	**0.034**	1.52	(1.13, 2.04)	**0.006**
				No	1.09	0.618	1.42	0.059	1.23	(0.96, 1.57)	0.099
*ABCC9*	Recessive	rs704178	G	Yes	1.77	0.121	1.48	0.171	1.58	(1.02, 2.47)	**0.042**
				No	1.33	0.292	1.43	0.180	1.38	(0.95, 2.01)	0.090
*APOE*	Additive	rs769449	A	Yes	1.15	0.589	1.54	0.094	1.33	(0.93, 1.90)	0.118
				No	1.30	0.188	2.02	**0.004**	1.54	(1.14, 2.09)	**0.005**
*APOE*	N/A	e4 Carrier	N/A	Yes	1.74	0.114	1.34	0.318	1.49	(0.96, 2.31)	0.075
				No	1.79	**0.024**	1.91	**0.018**	1.84	(1.28, 2.66)	**0.001**

Adjusted effects of single nucleotide variants (SNV) on hippocampal sclerosis (HS). A separate regression model was fit for each variant, mode of inheritance (MOI), and limbic-predominant age-related TDP-43 encephalopathy neuropathological change (LATE-NC) adjustment. All models also adjust for sex, age at death, first three principal components and cohort/study. For rs1990622, rs7781670, and rs704178, the effect alleles are the risk-associated alleles and not the minor alleles. NACC = National Alzheimer's Coordinating Center; ROSMAP = Religious Orders Study and Rush Memory and Aging Project; MOI = mode of inheritance; SNV = single-nucleotide variant; LATE-NC = limbic-predominant age-related TDP-43 encephalopathy neuropathological change; OR = odds ratio; CI = confidence interval.

An issue raised by the *ABCC9*/HS association results was whether this correlation was driven by cases lacking LATE-NC, i.e. the minority of cases with HS pathology that lacked TDP-43 proteinopathy. A separate sensitivity analysis was performed that excluded the cases with HS pathology that lacked LATE-NC. Results are shown in Additional File 1: Supplemental Table 3, which may be compared with Table 5. The odds ratio estimates for the association between *ABCC9* risk variants and HS pathology was essentially unchanged by removing the LATE-NC-HS + cases.

Additional sensitivity analyses were performed testing if the top LATE/HS-related SNVs were associated with ADNC, i.e. Braak NFT stages or CERAD neuritic plaque densities. In these analyses, only the *APOE* SNV (rs769449) and *APOE* ε4 carrier status were found to be associated with ADNC (Additional File 1: Supplemental Tables 7 and 8). These results suggest that the associations between HS and LATE-NC and the non-*APOE* SNVs were likely independent of ADNC.

## Discussion

Using large genetic data sets with complementary autopsy-derived data, we demonstrated that the neuropathological endophenotypes of LATE-NC and HS showed replication for associations with a number of previously identified risk genes. The strong association between *TMEM106B* and TDP-43 proteinopathy—including LATE-NC Stage 1—was once again replicated. Interestingly, *ABCC9* was not associated with LATE-NC but was associated with HS pathology. Our study adds to the growing body of literature on the overlapping genetics of HS and LATE-NC while also highlighting several genetic loci unique to each disease entity.

We replicated significant gene-based associations between HS and the *TMEM106B*, *ABCC9*, *GRN*, and *APOE* genes along with the rs7781670 (*TMEM106B*) and rs5848 (*GRN*) SNVs. Furthermore, we identified novel SNV-level associations between LATE-NC and rs7781670 and rs769449. The association of LATE-NC and rs7781670 is intriguing since it was also recently associated with clinical AD in a large AD GWAS [9]. We found no evidence to support the hypothesis that *KCNMB2* is a risk gene for either LATE-NC or HS pathologies. However, we note that the sample size of the present study was suitable to detect only relatively large genotype/phenotype associations.

There is an emerging consensus that mixed pathologies are highly prevalent in elderly populations, and there are complex relationships between genotypes and the downstream pathologies. The finding that variants in *GRN*, *TMEM106B*, and *APOE* genes are associated with several neuropathological endophenotypes fits in with recent studies looking at genetic pleiotropy in neurological conditions [12]. Pleiotropic effects have been observed among AD-related neuropathological changes like neuritic plaques, neurofibrillary tangles, and cerebral amyloid angiopathy [13] as well as between LATE-NC and FTLD-TDP [42].

Since the large majority of HS cases were also LATE-NC cases in the current study (Fig. 3), it was striking that some risk genes and SNVs were found to only be associated with HS and not LATE-NC—and vice versa, when statistical models were applied. We did identify several genes that are associated with both neuropathologic endophenotypes. Specifically, the *TMEM106B*, *GRN*, and *APOE* SNVs appear to predispose individuals to LATE-NC (Fig. 10a). Our data indicate that the associations between HS and SNVs in the *TMEM106B*, *GRN*, and *ABCC9* genes remain statistically significant in a model that adjusts for the presence of LATE-NC (Table 5). However, the impact of *TMEM106B* and *GRN* on HS appeared to be attenuated in a statistical model that included TDP-43 proteinopathy, suggesting that their impact on HS may be mediated by their role in LATE-NC. How these genetic SNVs can impact HS secondarily or independently of LATE-NC is not currently known.

While several *ABCC9* SNVs have been found to be associated with HS, including rs704178 and rs704180, this is the first study to report an association between the *ABCC9* SNV rs1914361 and HS. Notably, rs1914361 was found to be associated with HS in two of the three included cohorts of the original HS GWAS [43], but it was not included in the downstream analyses since its association with HS wasn’t nominally significant in all three cohorts (data not published). It is important to note that prior studies involved completely different sets of included participants (no overlap, as verified with computational methods) but the “direction” of the effect in all cohorts studied was the same. Since rs1914361 was found to also be significantly associated with the expression of *ABCC9* (Table 3) and is not in strong linkage disequilibrium with rs704178 (r^2^ = 0.176), the two loci may represent independent *ABCC9* HS risk SNVs.

**Fig. 9:**
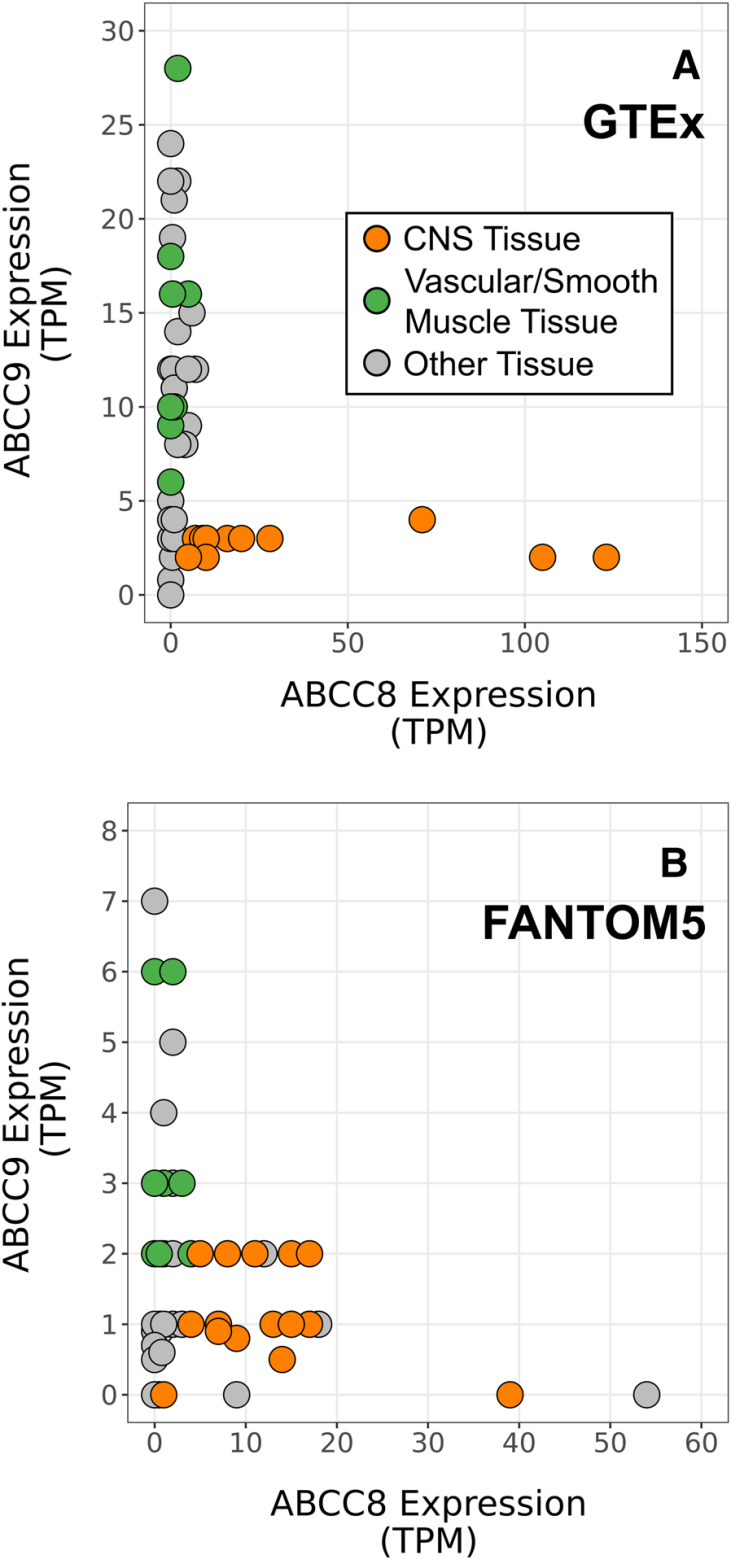
*ABCC8* and *ABCC9* gene expression across tissue types. *ABCC8* and *ABCC9* gene expression in various human tissues in the **a** Genotype-Tissue Expression (GTEx) and **b** Functional Annotation of Human Long Noncoding RNAs via Molecular Mapping (FANTOM5) databases. In GTEx, central nervous system (CNS) tissues included Brodmann (1909) area 24, Brodmann (1909) area 9, C1 segment of cervical spinal cord, amygdala, caudate nucleus, cerebellar hemisphere, cerebellum, cerebral cortex, hippocampus proper, hypothalamus, nucleus accumbens, pituitary gland, and substantia nigra; vascular/smooth muscle tissues included aorta, atrium auricular region, coronary artery, tibial artery, endocervix, esophagus muscularis mucosa, urinary bladder, and uterus; and other tissues included all other tissue types. In FANTOM5, CNS tissues included amygdala, brain, caudate nucleus, cerebellum, diencephalon, dorsal thalamus, globus pallidus, hippocampal formation, locus ceruleus, medulla oblongata, middle frontal gyrus, middle temporal gyrus, occipital cortex, occipital lobe, olfactory apparatus, parietal lobe, pituitary gland, putamen, spinal cord, and substantia nigra; vascular/smooth muscle tissue included artery, heart, heart left ventricle, left cardiac atrium, mitral valve, smooth muscle, tricuspid valve, and uterus; and other tissues included all other tissue types. GTEx = Genotype-Tissue Expression; FANTOM5 = Functional Annotation of Human Long Noncoding RNAs via Molecular Mapping; TPM = transcripts per million; CNS = central nervous system

We also identified divergent patterns in the tissue-level gene expressions of *ABCC9* and its homologous gene, *ABCC8* (Fig. 9). The proteins encoded by both of these genes function to help regulate the “KATP” potassium channels, which serve as molecular sensors helping to match metabolic needs with cellular reactivity [45, 51]. Multiple lines of evidence link *ABCC9* with blood vessels in normal and disease states. In the present study, *ABCC9* appeared to be relatively highly expressed in vascular and smooth muscle tissues (Fig. 9), and the correlative impact of the *ABCC9*/HS risk allele differed in blood vessels in comparison to brain tissue (Fig. 7). Further, the risk-related allele was associated with lower expression of *ABCC9* in blood vessels (Fig. 7). *ABCC9* has previously been shown to be a marker of vascular mural cells (e.g., pericytes and smooth muscle cells)[2, 5, 10, 30, 64] and the protein product has been implicated in modulating blood flow [45, 51, 61]. In terms of highly penetrant genetic variants, *ABCC9* toxic gain-of-function mutations are linked to Cantu Syndrome, a complex phenotype that includes tortuous cerebral blood vessel patterns [31, 32]. *ABCC9* loss-of-function mutations cause *ABCC9*-related Intellectual disability Myopathy Syndrome (AIMS), another complex condition that includes intellectual disability with white matter hyperintensities detected by MRI, even in teenagers [63]. Thus, *ABCC9* dysregulation may partly underlie the observation (i.e., may help to explain the phenomenon) that arteriolosclerosis is more severe in brains with LATE/HS than non-LATE/HS brains [1, 22, 24, 28, 49].

**Fig. 10:**
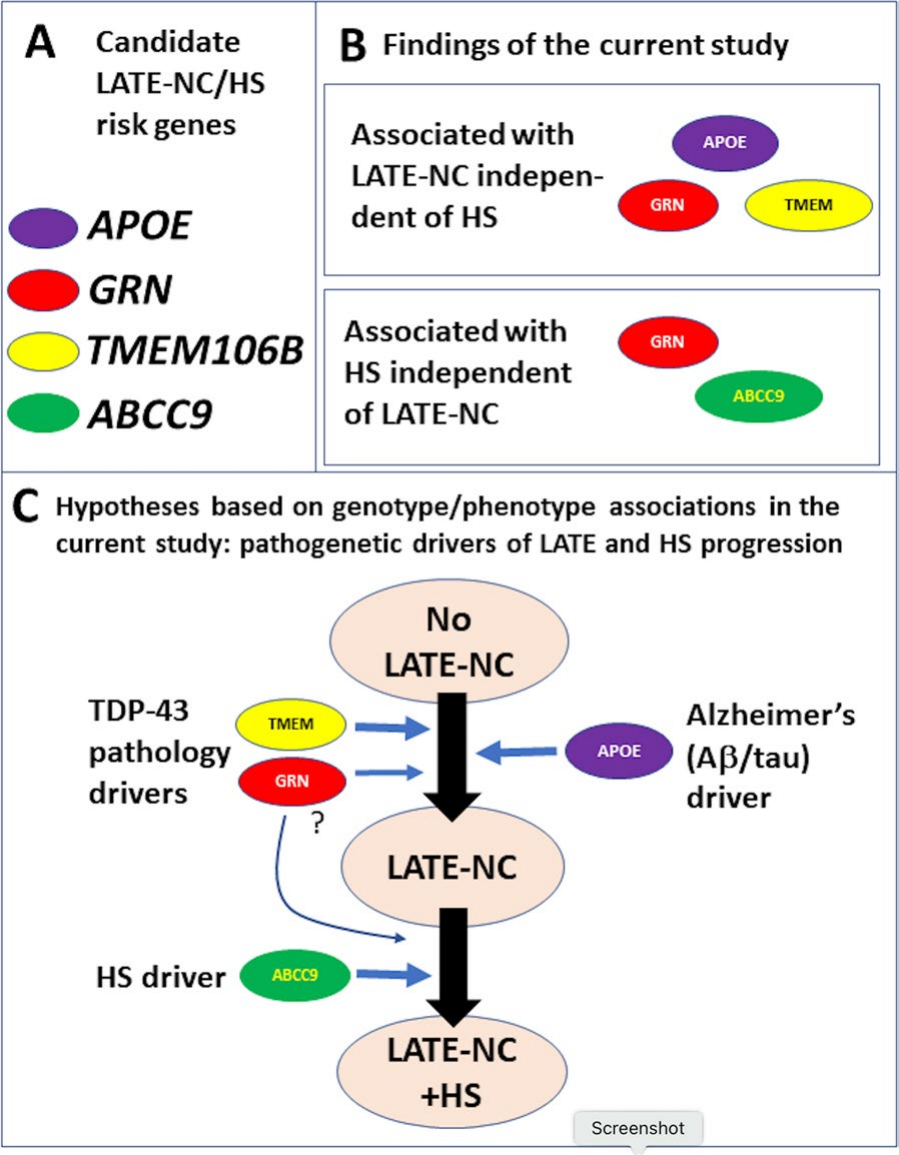
Diagrams depicting potential causal relationships between the genes under study with positive findings. Diagrams depicting potential causal relationships between the genes under study with positive findings (*TMEM106B*, *ABCC9*, *GRN*, and *APOE*) and TDP-43 proteinopathy/limbic-predominant age-related TDP-43 encephalopathy (LATE), hippocampal sclerosis (HS), and Alzheimer’s disease (AD). **a** The candidate genes and their corresponding colors in the diagrams, **b** a diagram of the current study’s prima facie results, and **c** a diagram showing hypothetical mechanistic pathways that are compatible with the findings of the current study, including how AD neuropathologic changes (often linked to the APOE risk allele) may fit in with the current study’s results. LATE = limbic-predominant age-related TDP-43 encephalopathy; HS = hippocampal sclerosis; AD = Alzheimer’s disease

The current study adds to a growing body of literature suggesting that LATE-NC is a potential precursor to HS [42]. It is yet to be seen how exactly the *APOE* gene and AD-type changes interact with other pathologies, but one hypothesis is that *APOE* and AD predispose an individual to LATE-NC, which then drives an individual towards severe LATE-NC and HS (Fig. 10). It has been found that TDP-43 proteinopathy localizes to tangle-like structures in many cases with ADNC [26]. Further autopsy-based studies with larger sample sizes are needed.

There are both limitations and strengths to the present study. Because of the characteristics of the sample (largely Caucasian, drawn from a number of different research centers), the degree to which findings are generalizable is unknown, especially with respect to individuals of other ancestries. While this work aims to replicate previous associations, there are many models considered which can inflate false positive rates. Additionally, it can be difficult to show that the associations identified in the current study are independent of ADNC, but several sensitivity analyses provide evidence that at least the non-*APOE* associations are likely independent of ADNC. Further follow-up studies are needed to investigate the significant associations between *APOE* and LATE-NC, though even if null this association would still highlight the strong associations that exist between AD and other neurodegenerative diseases, which is interesting in itself. We also note that all the included subjects had high-quality neuropathologic workup for TDP-43 proteinopathy and HS, and all the ADGC subjects were autopsied during 2014 and later. These study design elements constitute strengths of the current study.

## Supplementary Information

**Additional file 1. Supplemental Table 1** for a summary of the rare conditions excluded from the NACC sample; these conditions are extremely rare among ROSMAP participants. ROSMAP participants included in the Nelson et al. 2014 hippocampal sclerosis (HS) genome wise association study (GWAS) were explicitly excluded from the current study; NACC participants were only included in the current study if version 10 NACC neuropathology (NP) data were available, which were not collected until after 2014. LATE-NC = limbic-predominant age-related TDP-43 encephalopathy neuropathological change; HS = hippocampal sclerosis; NACC = National Alzheimer's Coordinating Center; ROSMAP = Religious Orders Study and Rush Memory and Aging Project; GWAS = genome wide association study.

## Data Availability

NACC data are available upon request via NACC’s website (https://naccdata.org/). ROSMAP data are available online at the Rush Alzheimer’s Disease Center Resource Sharing Hub (https://www.radc.rush.edu/), as well as on the Accelerating Medicines Partnership-Alzheimer’s Disease (AMP-AD) Knowledge Portal (syn3219045). ADGC data are available upon request via the NIAGADS website (https://www.niagads.org/user/login?destination=data/request/new_request/).

## Abbreviations

ACAT: Aggregated Cauchy association test
AD: Alzheimer’s disease
ADGC: Alzheimer’s disease genetics consortium
ADNC: Alzheimer’s disease neuropathological changes
ADRD: Alzheimer’s disease and related dementias
ALS: Amyotrophic lateral sclerosis
AMP-AD: Accelerating Medicines Partnership-Alzheimer’s Disease
eQTL: Expression quantitative trait loci
TLD: Frontotemporal lobar degeneration
FTLD-TDP: Frontotemporal lobe degeneration with TDP-43
GTEx: Genotype-Tissue Expression
GWAS: Genome wide association study
HS: Hippocampal sclerosis
HWE: Hardy–Weinberg equilibrium
IRB: Institutional Review
LATE: Limbic-predominant age-related TDP-43 encephalopathy
LATE-NC: Limbic-predominant age-related TDP-43 encephalopathy neuropathological changes
MOI: Mode of inheritance
NACC: National Alzheimer’s Coordinating Center
NP: Neuropathology
OR: Odds ratio
PC: Principal component
QC: Quality control
ROSMAP: Religious Orders Study and the Rush Memory and Aging Project
SNV: Single-nucleotide variant
TDP-43: TAR-DNA binding protein 43
