# Supplementary material for: Analysis of genes (TMEM106B, GRN, ABCC9, KCNMB2, and APOE) implicated in risk for LATE-NC and hippocampal sclerosis provides pathogenetic insights: a retrospective genetic association study
Source: Acta Neuropathol Commun. 2021 Sep 15;9:152. doi: 10.1186/s40478-021-01250-2 (PMC8442328; doi:10.1186/s40478-021-01250-2)
Supplement: Supplementary file 1 — Additional file 1. Supplemental Table 1 for a summary of the rare conditions excluded from the NACC sample; these conditions are extremely rare among ROSMAP participants. ROSMAP participants included in the Nelson et al. 2014 hippocampal sclerosis (HS) genome wise association study (GWAS) were explicitly excluded from the current study; NACC participants were only included in the current study if version 10 NACC neuropathology (NP) data were available, which were not collected until after 2014. LATE-NC = limbic-predominant age-related TDP-43 encephalopathy neuropathological change; HS = hippocampal sclerosis; NACC = National Alzheimer's Coordinating Center; ROSMAP = Religious Orders Study and Rush Memory and Aging Project; GWAS = genome wide association study. [file 40478_2021_1250_MOESM1_ESM.docx]

**Supplemental Tables**

**Supplemental Table 1**: Exclusion criteria in the National Alzheimer’s Coordination Center Neuropathology (NACC NP) dataset.

| **Exclusion criteria** | **Variable name** |
| --- | --- |
| Down syndrome | NACCDOWN |
| Pigment-spheroid degeneration/NBIA | NPPDXA |
| Multiple system atrophy | NPPDXB |
| Trinucleotide disease (Huntington disease, SCA, other) | NPPDXD |
| Malformation of cortical development | NPPDXE |
| Metabolic/storage disorder of any type | NPPDXF |
| White matter disease, leukodystrophy | NPPDXG |
| White matter disease, multiple sclerosis or other demyelinating disease | NPPDXH |
| Contusion/traumatic brain injury of any type, acute | NPPDXI |
| Contusion/traumatic brain injury of any type, chronic | NPPDXJ |
| Neoplasm, primary | NPPDXK |
| Neoplasm, metastatic | NPPDXL |
| Infectious process of any type (encephalitis, abscess, etc.) | NPPDXM |
| Herniation, any site | NPPDXN |
| Prion disease | NACCPRIO |
| FTLD-tau | NPFTDTAU |
| ALS/motor neuron disease (MND) | NPALSMND |
| CADASIL | NPPATH10 |
| Other FTLD* | NPOFTD |

*Other FTLD includes atypical FTLD-U, neuronal intermediate filament inclusions disease (NIFID), basophilic inclusion body disease (BIBD), ubiquitin-proteasome system (FTLD-UPS), dementia lacking distinctive histology and FTLD with no inclusions detected by tau, TDP-43, or ubiquitin.p62 IHC (FTLD-NOS).

**Supplemental Table 2**: GRCh37/hg19 gene boundaries and the corresponding number of variants included in each analysis.

| **Gene** | **Chr.** | **Canonical Transcript** | | **Canonical Transcript ± 10kb** | | | | |
| --- | --- | --- | --- | --- | --- | --- | --- | --- |
|  |  |  |  | **Start Pos.** | **End Pos.** | **No. of Variants Included in Analyses** | | |
|  |  | **Start Pos.** | **End Pos.** |  |  | **NACC** | **ROSMAP** | **Meta-Analysis** |
| *KCNMB2* | 3 | 178254085 | 178562217 | 178244085 | 178572217 | 955 | 953 | 929 |
| *TMEM106B* | 7 | 12250847 | 12276890 | 12240847 | 12286890 | 255 | 254 | 250 |
| *ABCC9* | 12 | 21950323 | 22089628 | 21940323 | 22099628 | 292 | 268 | 265 |
| *GRN* | 17 | 42422490 | 42430470 | 42412490 | 42440470 | 40 | 35 | 33 |
| *APOE* | 19 | 45409038 | 45412650 | 45399038 | 45422650 | 38 | 22 | 15 |

Chr. = chromosome; NACC = National Alzheimer's Coordinating Center; ROSMAP = Religious Orders Study and Rush Memory and Aging Project; MOI = mode of inheritance.

**Supplemental Table 3**: Sensitivity analyses for adjusted hippocampal sclerosis (HS) odds ratios.

| **Dataset** | **Subset** | **SNP** | **Effect Allele** | **MOI** | **No. of Obs.** | **OR** | **Lower 95% CL** | **Upper 95% CL** | **P-value** |
| --- | --- | --- | --- | --- | --- | --- | --- | --- | --- |
| NACC | All Available Observations | rs1914361 | G | Additive | 410 | 1.76 | 1.19 | 2.64 | 0.005051 |
| NACC | No HS+/LATE- Cases | rs1914361 | G | Additive | 393 | 1.81 | 1.16 | 2.87 | 0.010197 |
| NACC | All Available Observations | rs1914361 | G | Recessive | 410 | 2.85 | 1.52 | 5.28 | 0.000941 |
| NACC | No HS+/LATE- Cases | rs1914361 | G | Recessive | 393 | 2.61 | 1.26 | 5.25 | 0.007787 |
| NACC | All Available Observations | rs704178 | C | Additive | 410 | 0.74 | 0.49 | 1.11 | 0.151609 |
| NACC | No HS+/LATE- Cases | rs704178 | C | Additive | 393 | 0.8 | 0.5 | 1.28 | 0.363251 |
| NACC | All Available Observations | rs704178 | C | Recessive | 410 | 0.65 | 0.32 | 1.25 | 0.215645 |
| NACC | No HS+/LATE- Cases | rs704178 | C | Recessive | 393 | 0.75 | 0.33 | 1.53 | 0.445006 |
| ROSMAP | All Available Observations | rs1914361 | G | Additive | 732 | 1.38 | 0.97 | 1.97 | 0.078816 |
| ROSMAP | No HS+/LATE- Cases | rs1914361 | G | Additive | 727 | 1.5 | 1.04 | 2.19 | 0.031805 |
| ROSMAP | All Available Observations | rs1914361 | G | Recessive | 732 | 1.75 | 1 | 3 | 0.045225 |
| ROSMAP | No HS+/LATE- Cases | rs1914361 | G | Recessive | 727 | 1.99 | 1.12 | 3.46 | 0.016041 |
| ROSMAP | All Available Observations | rs704178 | C | Additive | 732 | 0.67 | 0.46 | 0.96 | 0.03165 |
| ROSMAP | No HS+/LATE- Cases | rs704178 | C | Additive | 727 | 0.66 | 0.45 | 0.97 | 0.035417 |
| ROSMAP | All Available Observations | rs704178 | C | Recessive | 732 | 0.5 | 0.22 | 0.98 | 0.059765 |
| ROSMAP | No HS+/LATE- Cases | rs704178 | C | Recessive | 727 | 0.47 | 0.2 | 0.96 | 0.055162 |

Sensitivity analyses showing the adjusted effects of single nucleotide variants (SNV) on hippocampal sclerosis (HS), excluding all LATE-NC-HS+ cases. All analyses were adjusted for sex, age at death, cohort/study, and the first three genetic principal components.

**Supplemental Table 4:** Additional participant characteristics for National Alzheimer’s Coordinating Center (NACC) participants stratified by combined limbic predominant age-related TDP-43 encephalopathy neuropathologic changes (LATE-NC) and hippocampal sclerosis (HS) case status.

|  | **Overall** | **Stratified by Combined LATE-NC and HS Case Status** | | | | |
| --- | --- | --- | --- | --- | --- | --- |
|  |  | **LATE- HS-** | **LATE+ HS-** | **LATE- HS+** | **LATE+ HS+** | **Unable to Ascertain** |
| **Number of Participants** | 633 | 273 | 72 | 17 | 48 | 223 |
| **Duration of symptoms, Mean (SD)** | 10.8 (4.9) | 10.5 (4.8) | 12.1 (4.8) | 12.0 (4.5) | 13.1 (3.7) | 9.9 (5.0) |
| **HS details, N (%)** |  |  |  |  |  |  |
| None | 543 (85.8) | 273 (100.0) | 72 (100.0) | 0 (0.0) | 0 (0.0) | 198 (88.8) |
| Unilateral | 8 (1.3) | 0 (0.0) | 0 (0.0) | 1 (5.9) | 6 (12.5) | 1 (0.4) |
| Bilateral | 16 (2.5) | 0 (0.0) | 0 (0.0) | 2 (11.8) | 13 (27.1) | 1 (0.4) |
| Present but laterality not assessed | 65 (10.3) | 0 (0.0) | 0 (0.0) | 14 (82.4) | 29 (60.4) | 22 (9.9) |
| Not assessed | 1 (0.2) | 0 (0.0) | 0 (0.0) | 0 (0.0) | 0 (0.0) | 1 (0.4) |

Participant characteristics stratified by combined limbic-predominant age-related TDP-43 encephalopathy neuropathological changes (LATE-NC) and hippocampal sclerosis (HS) case status. Participants missing data for either LATE-NC or HS are labeled as “unable to ascertain.” NACC = National Alzheimer's Coordinating Center; SD = standard deviation; HS = hippocampal sclerosis; LATE-NC = limbic-predominant age-related TDP-43 encephalopathy neuropathological changes.

**Supplemental Table 5:** Participant characteristics and concomitant neuropathologies for National Alzheimer's Coordinating Center (NACC) and Religious Orders Study and Rush Memory and Aging Project (ROSMAP) participants.

|  | **NACC** | | | | | | **ROSMAP** | | | | | |
| --- | --- | --- | --- | --- | --- | --- | --- | --- | --- | --- | --- | --- |
|  | **Overall** | **LATE- HS-** | **LATE+ HS-** | **LATE- HS+** | **LATE+ HS+** | **Inconclusive** | **Overall** | **LATE- HS-** | **LATE+ HS-** | **LATE- HS+** | **LATE+ HS+** | **Inconclusive** |
| **Number of Participants** | 633 | 273 | 72 | 17 | 48 | 223 | 795 | 485 | 176 | 5 | 66 | 63 |
| **Age at death, Mean (SD)** | 85.9 (8.3) | 85.0 (8.0) | 85.4 (7.4) | 84.8 (9.1) | 85.4 (7.4) | 87.4 (8.8) | 88.7 (7.2) | 87.9 (7.2) | 91.0 (6.2) | 83.3 (9.6) | 92.6 (5.8) | 85.2 (7.3) |
| **Female, N (%)** | 320 (50.6) | 129 (47.3) | 40 (55.6) | 8 (47.1) | 29 (60.4) | 114 (51.1) | 534 (67.2) | 307 (63.3) | 134 (76.1) | 4 (80.0) | 52 (78.8) | 37 (58.7) |
| **Braak stage V/VI, N (%)** |  |  |  |  |  |  |  |  |  |  |  |  |
| No | 243 (38.4) | 123 (45.1) | 11 (15.3) | 5 (29.4) | 11 (22.9) | 93 (41.7) | 576 (72.5) | 382 (78.8) | 105 (59.7) | 2 (40.0) | 36 (54.5) | 51 (81.0) |
| Yes | 387 (61.1) | 150 (54.9) | 60 (83.3) | 12 (70.6) | 36 (75.0) | 129 (57.8) | 219 (27.5) | 103 (21.2) | 71 (40.3) | 3 (60.0) | 30 (45.5) | 12 (19.0) |
| Unknown | 3 (0.5) | 0 (0.0) | 1 (1.4) | 0 (0.0) | 1 (2.1) | 1 (0.4) | 0 (0.0) | 0 (0.0) | 0 (0.0) | 0 (0.0) | 0 (0.0) | 0 (0.0) |
| **CERAD C3, N (%)** |  |  |  |  |  |  |  |  |  |  |  |  |
| No | 324 (51.2) | 144 (52.7) | 22 (30.6) | 9 (52.9) | 14 (29.2) | 135 (60.5) | 537 (67.5) | 359 (74.0) | 102 (58.0) | 2 (40.0) | 30 (45.5) | 44 (69.8) |
| Yes | 307 (48.5) | 128 (46.9) | 50 (69.4) | 8 (47.1) | 34 (70.8) | 87 (39.0) | 258 (32.5) | 126 (26.0) | 74 (42.0) | 3 (60.0) | 36 (54.5) | 19 (30.2) |
| Unknown | 2 (0.3) | 1 (0.4) | 0 (0.0) | 0 (0.0) | 0 (0.0) | 1 (0.4) | 0 (0.0) | 0 (0.0) | 0 (0.0) | 0 (0.0) | 0 (0.0) | 0 (0.0) |
| **Moderate/severe B-ASC, N (%)** |  |  |  |  |  |  |  |  |  |  |  |  |
| No | 297 (46.9) | 143 (52.4) | 19 (26.4) | 8 (47.1) | 15 (31.2) | 112 (50.2) | 568 (71.4) | 359 (74.0) | 124 (70.5) | 4 (80.0) | 41 (62.1) | 40 (63.5) |
| Yes | 310 (49.0) | 129 (47.3) | 53 (73.6) | 9 (52.9) | 33 (68.8) | 86 (38.6) | 227 (28.6) | 126 (26.0) | 52 (29.5) | 1 (20.0) | 25 (37.9) | 23 (36.5) |
| Unknown | 26 (4.1) | 1 (0.4) | 0 (0.0) | 0 (0.0) | 0 (0.0) | 25 (11.2) | 0 (0.0) | 0 (0.0) | 0 (0.0) | 0 (0.0) | 0 (0.0) | 0 (0.0) |
| **Lewy Bodies, N (%)** |  |  |  |  |  |  |  |  |  |  |  |  |
| No | 421 (66.5) | 188 (68.9) | 41 (56.9) | 10 (58.8) | 23 (47.9) | 159 (71.3) | 609 (76.6) | 382 (78.8) | 125 (71.0) | 2 (40.0) | 66 (59.1) | 51 (81.0) |
| Yes | 208 (32.9) | 85 (31.1) | 31 (43.1) | 6 (35.3) | 25 (52.1) | 61 (27.4) | 186 (24.2) | 103 (21.9) | 46 (26.2) | 2 (40.0) | 23 (34.8) | 12 (19.0) |
| Unknown | 4 (0.6) | 0 (0.0) | 0 (0.0) | 1 (5.9) | 0 (0.0) | 3 (1.3) | 0 (0.0) | 0 (0.0) | 5 (2.8) | 1 (20.0) | 4 (6.1) | 0 (0.0) |

Participant characteristics stratified by combined limbic-predominant age-related TDP-43 encephalopathy neuropathological changes (LATE-NC) and hippocampal sclerosis (HS) case status. Participants missing data for either LATE-NC or HS are labeled as “inconclusive.” NACC = National Alzheimer's Coordinating Center; ROSMAP = Religious Orders Study and Rush Memory and Aging Project; SD = standard deviation; HS = hippocampal sclerosis; LATE-NC = limbic-predominant age-related TDP-43 encephalopathy neuropathological changes; B-ASC = brain arteriolosclerosis.

**Supplemental Table 6:** Adjusted limbic predominant age-related TDP-43 encephalopathy (LATE) Stage 1 (vs. LATE Stage 0) odds ratios for risk variants.

| **Gene** | **MOI** | **SNV** | **Effect Allele** | **NACC** | | **ROSMAP** | | **Meta-Analysis** | | |
| --- | --- | --- | --- | --- | --- | --- | --- | --- | --- | --- |
|  |  |  |  | **OR** | **P-value** | **OR** | **P-value** | **OR** | **95% CI** | **P-value** |
| *TMEM106B* | Additive | rs7781670 | G | 1.65 | 0.192 | 1.26 | 0.121 | 1.30 | 0.99 - 1.70 | 0.055 |
| *TMEM106B* | Additive | rs1990622 | G | 1.94 | 0.088 | 1.29 | 0.082 | **1.36** | **1.04 - 1.78** | **0.026** |
| *GRN* | Additive | rs5848 | T | 1.30 | 0.474 | 1.11 | 0.537 | 1.14 | 0.85 - 1.54 | 0.391 |
| *ABCC9* | Additive | rs1914361 | G | 1.32 | 0.414 | 1.19 | 0.261 | 1.21 | 0.92 - 1.59 | 0.174 |
| *ABCC9* | Recessive | rs1914361 | G | 0.90 | 0.859 | 1.33 | 0.258 | 1.26 | 0.80 - 1.98 | 0.326 |
| *ABCC9* | Additive | rs704178 | C | 0.98 | 0.947 | 0.96 | 0.793 | 0.96 | 0.73 - 1.27 | 0.789 |
| *ABCC9* | Recessive | rs704178 | C | 0.87 | 0.818 | 0.97 | 0.892 | 0.95 | 0.60 - 1.51 | 0.830 |
| *APOE* | Additive | rs769449 | A | 0.78 | 0.564 | 1.67 | 0.032 | 1.39 | 0.92 - 2.09 | 0.114 |
| *APOE* | N/A | ε4 Carrier | N/A | 0.90 | 0.778 | 1.53 | 0.065 | 1.33 | 0.90 - 1.97 | 0.149 |

Adjusted effects of single nucleotide variants (SNV) on limbic predominant age-related TDP-43 encephalopathy (LATE) Stage 1 (vs. LATE Stage 0). A separate regression model was fit for each variant and mode of inheritance (MOI). All models also adjust for sex, age at death, first three principal components, and cohort/study. NACC = National Alzheimer's Coordinating Center; ROSMAP = Religious Orders Study and Rush Memory and Aging Project; MOI = mode of inheritance; SNV = single-nucleotide variant; LATE-NC = limbic-predominant age-related TDP-43 encephalopathy neuropathological change; OR = odds ratio; CI = confidence interval.

**Supplemental Table 7:** Adjusted neurofibrillary tangles (Braak NFT Stages V/VI) odds ratios for risk variants.

| **Gene** | **MOI** | **SNV** | **Effect Allele** | **NACC** | | **ROSMAP** | | **Meta-Analysis** | | |
| --- | --- | --- | --- | --- | --- | --- | --- | --- | --- | --- |
|  |  |  |  | **OR** | **P-value** | **OR** | **P-value** | **OR** | **95% CI** | **P-value** |
| *TMEM106B* | Additive | rs7781670 | G | 0.98 | 0.910 | 1.17 | 0.177 | 1.09 | 0.92 - 1.30 | 0.331 |
| *TMEM106B* | Additive | rs1990622 | G | 0.97 | 0.830 | 1.16 | 0.194 | 1.08 | 0.91 - 1.29 | 0.390 |
| *GRN* | Additive | rs5848 | T | 0.88 | 0.389 | 0.81 | 0.105 | 0.84 | 0.69 - 1.02 | 0.076 |
| *ABCC9* | Additive | rs1914361 | G | 1.07 | 0.600 | 1.07 | 0.564 | 1.07 | 0.90 - 1.28 | 0.436 |
| *ABCC9* | Recessive | rs1914361 | G | 1.19 | 0.486 | 1.07 | 0.740 | 1.11 | 0.82 - 1.51 | 0.488 |
| *ABCC9* | Additive | rs704178 | C | 1.03 | 0.820 | 1.02 | 0.867 | 1.03 | 0.86 - 1.22 | 0.783 |
| *ABCC9* | Recessive | rs704178 | C | 1.11 | 0.626 | 1.04 | 0.854 | 1.07 | 0.80 - 1.43 | 0.642 |
| *APOE* | Additive | rs769449 | A | 2.76 | 2.221 x 10^-7^ | 2.31 | 1.691 x 10^-6^ | **2.50** | **1.93 - 3.23** | **2.212 x 10^-12^** |
| *APOE* | N/A | ε4 Carrier | N/A | 3.82 | 1.561 x 10^-10^ | 2.84 | 2.367 x 10^-8^ | **3.24** | **2.46 - 4.26** | **3.580 x 10^-17^** |

Adjusted effects of single nucleotide variants (SNV) on neurofibrillary tangles. Neurofibrillary tangles were defined as a Braak stage of V or VI. A separate regression model was fit for each variant and mode of inheritance (MOI). All models also adjust for sex, age at death, first three principal components, and cohort/study. NACC = National Alzheimer's Coordinating Center; ROSMAP = Religious Orders Study and Rush Memory and Aging Project; MOI = mode of inheritance; SNV = single-nucleotide variant; LATE-NC = limbic-predominant age-related TDP-43 encephalopathy neuropathological change; OR = odds ratio; CI = confidence interval.

**Supplemental Table 8:** Adjusted frequent neuritic plaque odds ratios for risk variants.

| **Gene** | **MOI** | **SNV** | **Effect Allele** | **NACC** | | **ROSMAP** | | **Meta-Analysis** | | |
| --- | --- | --- | --- | --- | --- | --- | --- | --- | --- | --- |
|  |  |  |  | **OR** | **P-value** | **OR** | **P-value** | **OR** | **95% CI** | **P-value** |
| *TMEM106B* | Additive | rs7781670 | G | 0.89 | 0.389 | 0.90 | 0.319 | 0.89 | 0.75 - 1.06 | 0.188 |
| *TMEM106B* | Additive | rs1990622 | G | 0.84 | 0.212 | 0.92 | 0.436 | 0.89 | 0.75 - 1.05 | 0.165 |
| *GRN* | Additive | rs5848 | T | 0.78 | 0.086 | 0.99 | 0.902 | 0.89 | 0.75 - 1.07 | 0.228 |
| *ABCC9* | Additive | rs1914361 | G | 1.16 | 0.278 | 1.07 | 0.548 | 1.10 | 0.93 - 1.31 | 0.248 |
| *ABCC9* | Recessive | rs1914361 | G | 1.44 | 0.137 | 1.03 | 0.860 | 1.17 | 0.87 - 1.57 | 0.297 |
| *ABCC9* | Additive | rs704178 | C | 1.08 | 0.549 | 0.95 | 0.636 | 1.00 | 0.85 - 1.19 | 0.984 |
| *ABCC9* | Recessive | rs704178 | C | 1.10 | 0.650 | 0.94 | 0.728 | 1.01 | 0.76 - 1.33 | 0.962 |
| *APOE* | Additive | rs769449 | A | 1.98 | 3.805 x 10^-5^ | 2.84 | 8.477 x 10^-10^ | **2.36** | **1.87 - 2.98** | **4.664 x 10^-13^** |
| *APOE* | N/A | ε4 Carrier | N/A | 2.36 | 1.150 x 10^-5^ | 3.09 | 1.819 x 10^-10^ | **2.74** | **2.12 - 3.54** | **1.712 x 10^-14^** |

Adjusted effects of single nucleotide variants (SNV) on frequent neuritic plaques. A separate regression model was fit for each variant and mode of inheritance (MOI). All models also adjust for sex, age at death, first three principal components, and cohort/study. NACC = National Alzheimer's Coordinating Center; ROSMAP = Religious Orders Study and Rush Memory and Aging Project; MOI = mode of inheritance; SNV = single-nucleotide variant; LATE-NC = limbic-predominant age-related TDP-43 encephalopathy neuropathological change; OR = odds ratio; CI = confidence interval.

**Supplemental Figure**

**Supplemental Figure 1:** Variant-level results for *KCNMB2*.


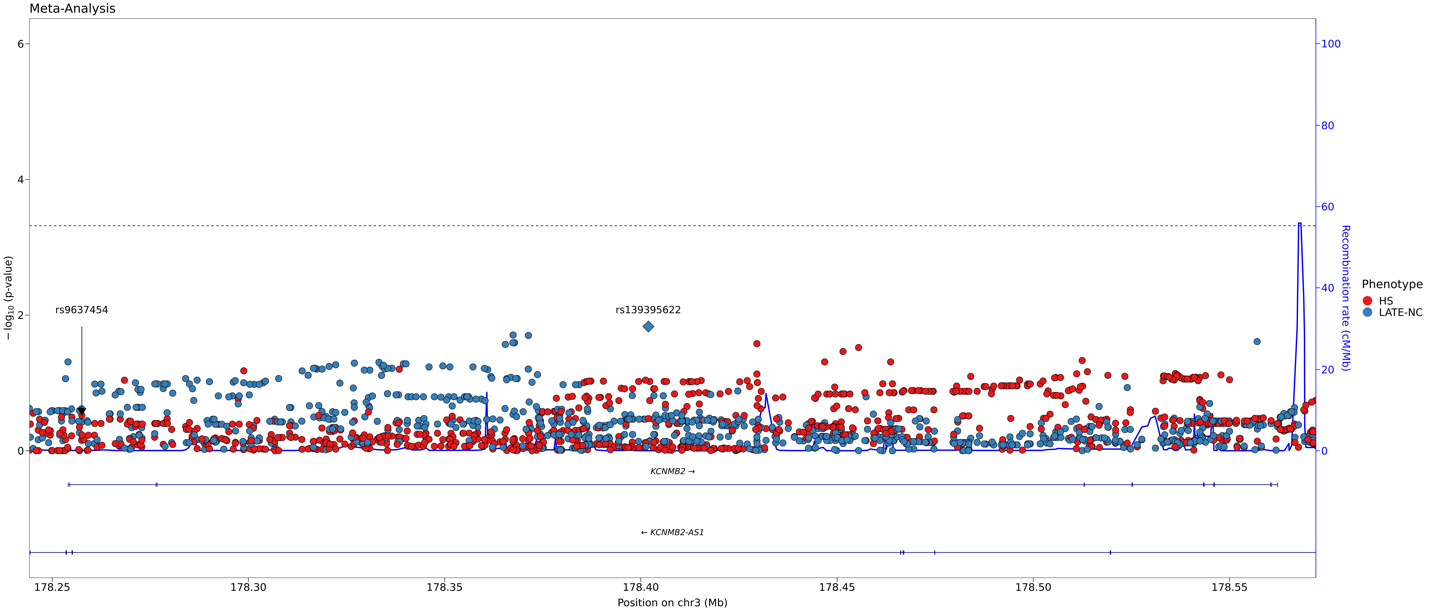


Adjusted, meta-analytic, single nucleotide variant (SNV)-level p-values for hippocampal sclerosis (HS) and limbic-predominant age-related TDP-43 encephalopathy neuropathological change (LATE-NC) across KCNMB2 ± 10kb. All analyses were adjusted for sex, age at death, cohort/study, and the first three genetic principal components. The horizontal dashed line represents the Bonferroni-corrected threshold for significance that accounts for the number of independent tests in the KCNMB2 ± 10kb region. A diamond represents the SNV with the smallest p-value. The previously identified KCNMB2 SNV (Beecham et al., 2014) is labeled and identified with an arrow. MOI = mode of inheritance; LATE-NC = limbic-predominant age-related TDP-43 encephalopathy neuropathological change; HS = hippocampal sclerosis.
